# Intracellular Zn^2+^ transients modulate global gene expression in dissociated rat hippocampal neurons

**DOI:** 10.1038/s41598-019-45844-2

**Published:** 2019-06-28

**Authors:** Lynn Sanford, Margaret C. Carpenter, Amy E. Palmer

**Affiliations:** Department of Biochemistry, BioFrontiers Institute, University of Colorado, Boulder, 80303 USA

**Keywords:** Gene expression analysis, Molecular neuroscience

## Abstract

Zinc (Zn^2+^) is an integral component of many proteins and has been shown to act in a regulatory capacity in different mammalian systems, including as a neurotransmitter in neurons throughout the brain. While Zn^2+^ plays an important role in modulating neuronal potentiation and synaptic plasticity, little is known about the signaling mechanisms of this regulation. In dissociated rat hippocampal neuron cultures, we used fluorescent Zn^2+^ sensors to rigorously define resting Zn^2+^ levels and stimulation-dependent intracellular Zn^2+^ dynamics, and we performed RNA-Seq to characterize Zn^2+^-dependent transcriptional effects upon stimulation. We found that relatively small changes in cytosolic Zn^2+^ during stimulation altered expression levels of 931 genes, and these Zn^2+^ dynamics induced transcription of many genes implicated in neurite expansion and synaptic growth. Additionally, while we were unable to verify the presence of synaptic Zn^2+^ in these cultures, we did detect the synaptic vesicle Zn^2+^ transporter ZnT3 and found it to be substantially upregulated by cytosolic Zn^2+^ increases. These results provide the first global sequencing-based examination of Zn^2+^-dependent changes in transcription and identify genes that may mediate Zn^2+^-dependent processes and functions.

## Introduction

Zinc (Zn^2+^) is an essential trace element that is increasingly suggested to play a signaling role in a variety of different cell types. Transient Zn^2+^ increases have been linked to many aspects of neuronal regulation and physiology^1–4^, pro-inflammatory signaling in monocytes^5^, oocyte maturation^6^, and modulation of Ca^2+^ release in cardiomyocytes^7^. In some cases, researchers have identified specific Zn^2+^-sensing proteins, such as neurotransmitter receptors or phosphatases^8–11^, or Zn^2+^-dependent regulation of signaling pathways, including Zn^2+^ modulation of the mitogen-activated protein kinase (MAPK) pathway^12,13^. However, there is still no unified mechanistic insight into how Zn^2+^ fluctuations induce changes in cellular physiology. Using dissociated hippocampal neurons as a model system, we present here the first global sequencing-based examination of potential downstream targets of Zn^2+^ signals.

Zn^2+^ is concentrated into glutamate-containing synaptic vesicles in subsets of neurons throughout the brain and is heavily enriched in the hippocampus^14^. Electrophysiology studies in brain slices have demonstrated that Zn^2+^ is released upon neuronal activation and modulates postsynaptic glutamate receptors^2,4,15^. Transient increases in Zn^2+^ have been observed inside neurons after stimulation, possibly as a result of translocation of Zn^2+^ from the synapse or release of Zn^2+^ from intracellular stores^16–18^. Both synaptic and intracellular Zn^2+^ signals contribute to regulation of short- and long-term plasticity in different areas of the brain^1,15,19,20^, and genetic or pharmacological manipulation of hippocampal Zn^2+^ leads to learning and memory deficits in rodents^21–23^. However, the cellular mechanisms underlying Zn^2+^-dependent neuronal remodeling are largely unclear.

In order to gain further insight into downstream effects of neuronal Zn^2+^ signaling, we performed RNA-Seq on dissociated hippocampal neuron cultures. While synaptic Zn^2+^ has been primarily characterized in brain slices or *in vivo* in rodent brains^15,24^, cultured neurons allow for uniform stimulation of a more homogenous population of cells than brain tissue. Furthermore, single-cell fluorescence imaging permits more robust quantification of intracellular Zn^2+^ dynamics than can be accomplished in tissue, giving us the ability to define a stimulation-induced Zn^2+^ signal and investigate its downstream consequences. The extent of synaptic Zn^2+^ mobilization in neuron cultures would inform any analysis of Zn^2+^-dependent global changes. Unfortunately, while dissociated hippocampal neurons have been shown to both release Zn^2+^ and exhibit cytosolic Zn^2+^ dynamics upon intensive glutamate stimulation^17,25^, no rigorous characterization of synaptic Zn^2+^ or synaptic Zn^2+^ machinery has been performed in these cultures.

In this work, we aimed to quantify cytosolic Zn^2+^ in dissociated hippocampal neurons under resting and mild stimulation conditions, characterize the synaptic Zn^2+^ pools in these neurons, and determine the transcriptional effects of cytosolic Zn^2+^ signals. We found that dissociated neurons accumulate the synaptic transporter ZnT3 (*Slc30a3*), responsible for loading Zn^2+^ into synaptic vesicles, although we were unable to confirm the presence of synaptic Zn^2+^ due to limitations of existing tools. However, we did find that stimulation of neurons with a short KCl treatment generated modest Zn^2+^ signals that increased 3-fold in the presence of low levels of exogenous Zn^2+^. Further, we observed robust Zn^2+^-dependent differential expression of 931 genes, many of which are related to neuronal physiology and synaptic modulation. To our knowledge, this is the first large-scale experiment to identify transcriptional changes of Zn^2+^ signals in a mammalian system, and these results can provide possible mechanistic insight into Zn^2+^-dependent neuronal plasticity.

## Results

### Characterization of hippocampal cultures as a model for Zn^2+^ signaling

**Figure 1:**
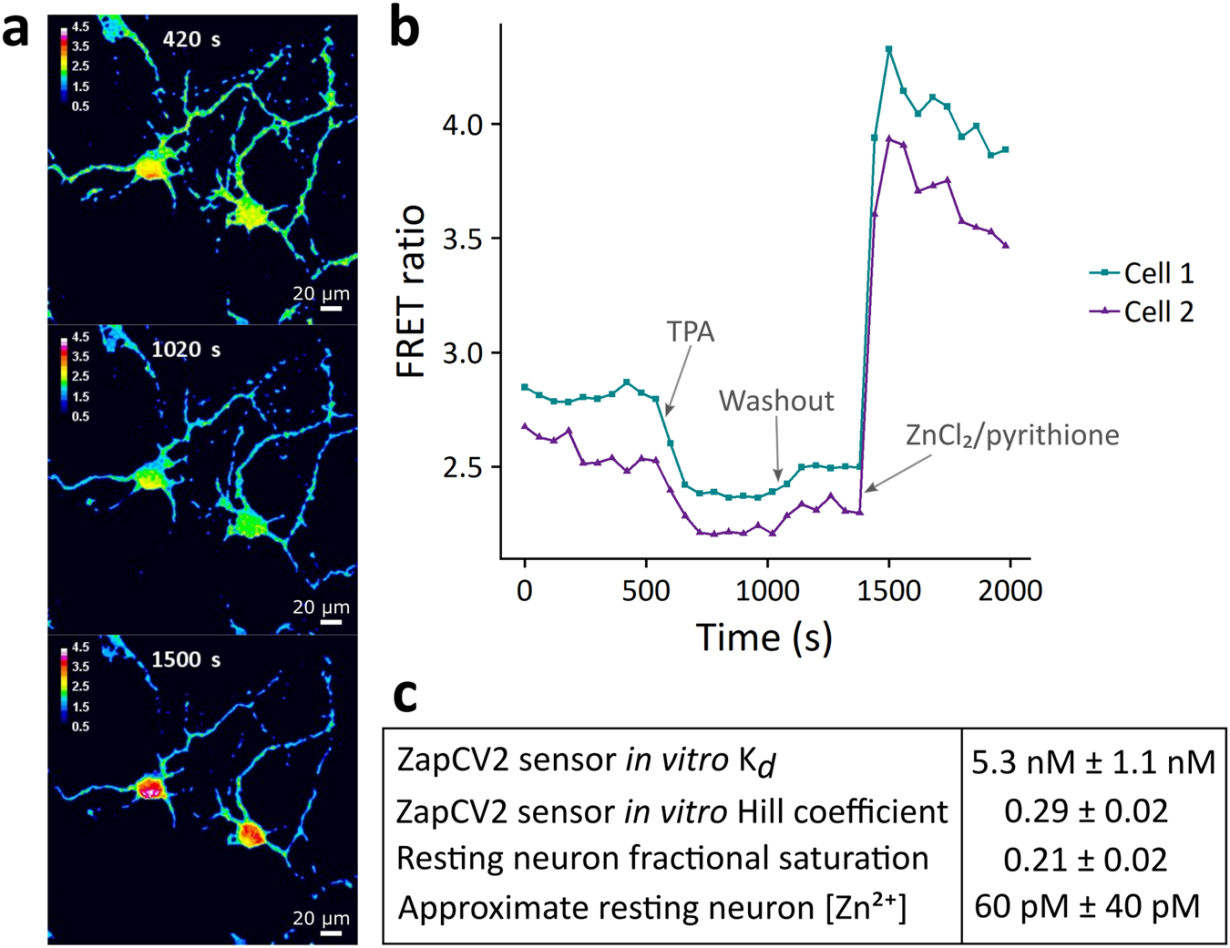
Genetically encoded Zn^2+^ FRET sensor measurements in resting neurons. (**a**) Images displaying the FRET ratio (FRET channel intensity/CFP channel intensity) of relevant neuronal areas within a field of view. Cells and times correspond to the traces in part (**b**) and picture resting (top), minimal (middle), and maximal (bottom) ratios obtained during a calibration. (**b**) Sample traces from a typical FRET sensor calibration. Each trace is derived from a region of interest in two separate cells within one field of view. Resting FRET ratios were observed, followed by treatment with 10 µM TPA and subsequently 10 µM ZnCl_2_/2.5 µM pyrithione to determine minimum and maximum FRET ratios, respectively. (**c**) Quantification of Zn^2+^ based on the *in vitro* binding parameters of the sensor. Errors correspond to standard error of the mean. n = 14 cells from 6 separate biological replicates derived from 2 separate cell preparations.

In order to rigorously define the overall Zn^2+^ status of dissociated hippocampal neuron cultures, we first characterized resting neuronal cytosolic and synaptic Zn^2+^. We used a genetically encoded Zn^2+^ Förster Resonance Energy Transfer (FRET) sensor to quantify cytosolic labile Zn^2+^ under resting conditions in neuronal cultures (Fig. 1a,b, Supplemental Fig. S1 and Supplemental Movie 1). We observed a resting fractional saturation for our sensor of 0.21 ± 0.02, which corresponds to an approximate Zn^2+^ concentration of 60 pM ± 40 pM (Fig. 1c). It should be noted that this estimate is based on an *in vitro* FRET sensor *K*_*d*_ of 5.3 nM (Fig. 1c and Supplemental Fig. S2), which may be altered in cells. Nevertheless, this Zn^2+^ concentration is comparable to that observed in other cell types^26,27^ and also is in accordance with previous measurements in neurons^27^.

**Figure 2:**
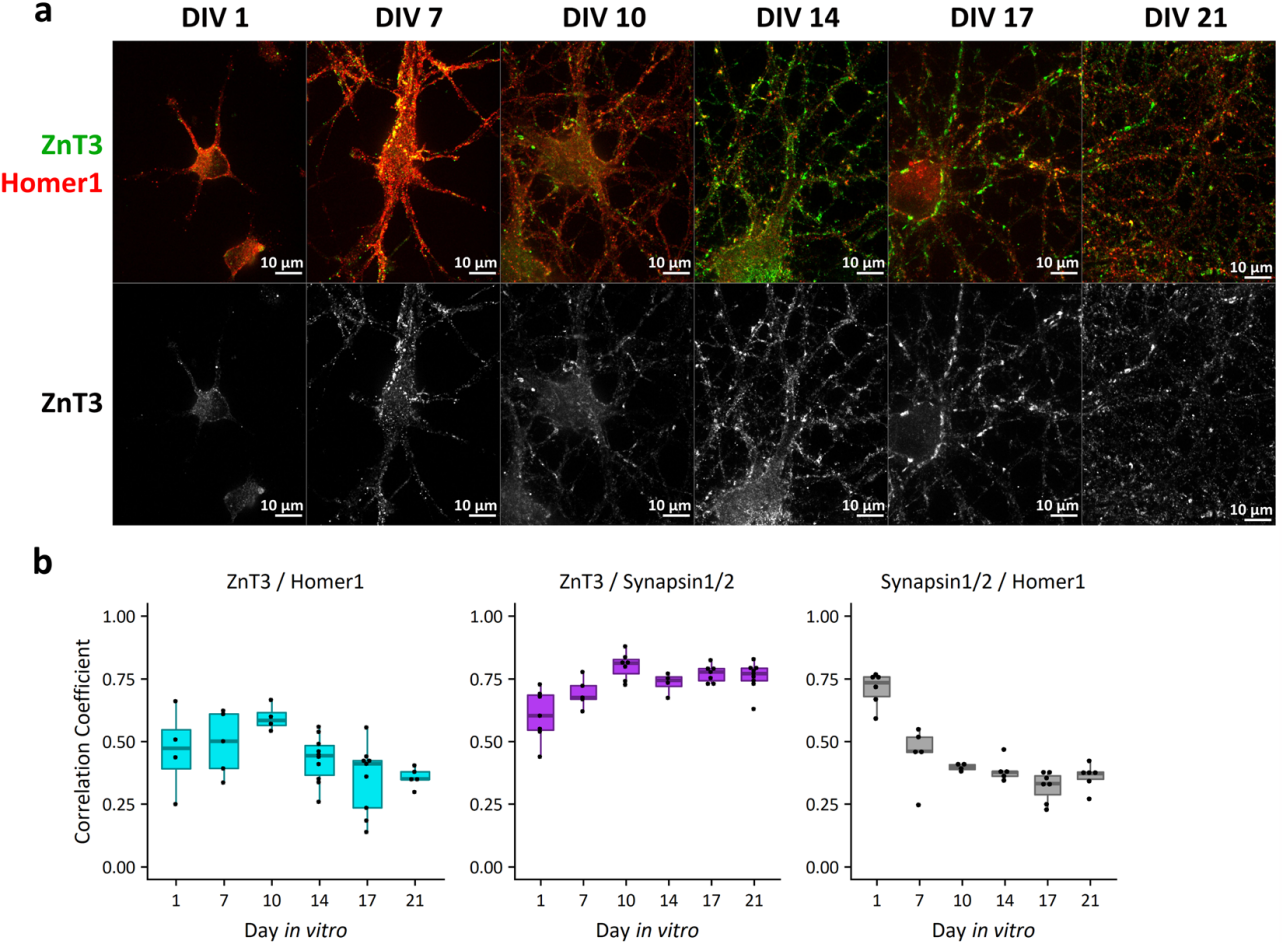
Expression and localization of Zn^2+^ transporter ZnT3 in cultured neurons by immunofluorescence. (**a**) ZnT3 (synaptic vesicle Zn^2+^ transporter, pseudocolored green or displayed in grayscale below) and Homer1 (post-synaptic density protein, pseudocolored red) are stained at different timepoints in culture. ZnT3 increases in expression over time in culture, and shows synaptic localization starting at DIV 10. Channel intensities of all images are scaled identically. (**b**) 2D correlation coefficients calculated on raw immunofluorescence images. Smaller correlation coefficients are more likely to represent less colocalization. ZnT3 maintains a high correlation coefficient compared to Synapsin1/2 across the time course, whereas Synapsin1/2 compared to Homer1 and ZnT3 compared to Homer1 decrease over time. Each DIV/protein comparison is one biological replicate, with each dot representing a separate field of view within the sample.

There are two main logical possibilities for how cytosolic Zn^2+^ signals can be generated. One is that intracellular, protein-bound stores of Zn^2+^ are mobilized upon stimulation. The second is that Zn^2+^ is released from synaptic vesicles into the synapse upon stimulation and subsequently translocates across the plasma membrane into the cytosol of postsynaptic or presynaptic neurons. The latter possibility depends upon the presence of ZnT3 and concentration of Zn^2+^ into synaptic vesicles. To determine whether dissociated neuron cultures have the machinery for synaptic vesicle Zn^2+^ sequestration, we investigated whether the synaptic vesicle Zn^2+^ transporter ZnT3 (*Slc30a3*) was expressed in cultures (Fig. 2, Supplemental Figs S3–S6). *Slc30a3* mRNA and synaptic Zn^2+^ are barely detectable in embryonic brain slices, with both increasing substantially after birth^24,28^. Accordingly, *Slc30a3* mRNA was not evident in our cells upon isolation (Day *in vitro* 0, DIV 0) as measured by RT-qPCR, but was present at all other timepoints (Supplemental Fig. S3). ZnT3 protein was observed by immunofluorescence, with robust expression after DIV 1 (Fig. 2a, Supplemental Fig. S4). By DIV 10, ZnT3 staining showed a synaptic pattern, colocalizing with presynaptic marker Synapsin1/2 and lying adjacent to postsynaptic protein Homer1 (Fig. 2b, Supplemental Fig. S5). Co-incubating samples with the ZnT3 antibody and a ZnT3-derived peptide abolished staining (Supplemental Fig. S6). This evidence indicates that ZnT3 is expressed and correctly localized in dissociated neuron culture.

**Figure 3:**
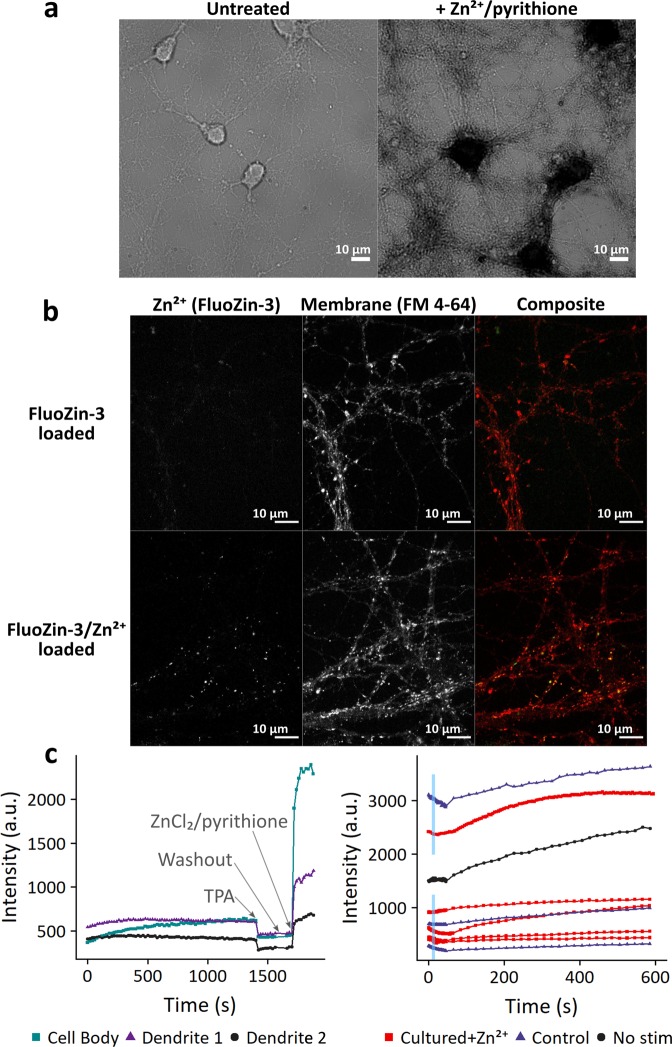
Attempts to visualize Zn^2+^ in synaptic vesicles. (**a**) Timm’s stain of neurons at DIV 10. Cultures were stained +/− treatment with 20 µM ZnCl_2_/2.5 µM pyrithione for 8 minutes prior to staining. Timm’s stain was visible in cell bodies and processes of treated neurons (right), but not visible under endogenous Zn^2+^ conditions (left). (**b**) Dye loading of stimulated neurons. Cultures were electrically stimulated in the presence of extracellular fluorescent dyes FluoZin-3 (10–50 µM, green) and FM 4–64 (5 µM, red), then washed to allow visualization of internalized dye. In some samples, 10 µM ZnCl_2_ was added to media before and during stimulation. FluoZin-3 puncta present upon co-incubation with Zn^2+^ indicate successful dye loading; however, no puncta are visible under endogenous Zn^2+^ conditions.

Following our observation that synaptic ZnT3 is present in hippocampal neuron cultures after DIV 10, we carried out a variety of experiments to investigate whether there was direct evidence for the presence or release of synaptic Zn^2+^ in these neurons (Fig. 3, Supplemental Figs S7–S8). In our first attempts, we applied a Timm stain, a histological stain that is commonly used to detect Zn^2+^ in brain tissue (Fig. 3a)^29–31^. We observed significant contrast, as evidenced by the dark staining due to sulfide/silver deposition, in neurons loaded with exogenous Zn^2+^ (Fig. 3a, Zn^2+^/pyrithione treatment). However, we saw no clear signal corresponding to endogenous Zn^2+^ in the cell body or in processes (Fig. 3a, untreated).

**Figure 4:**
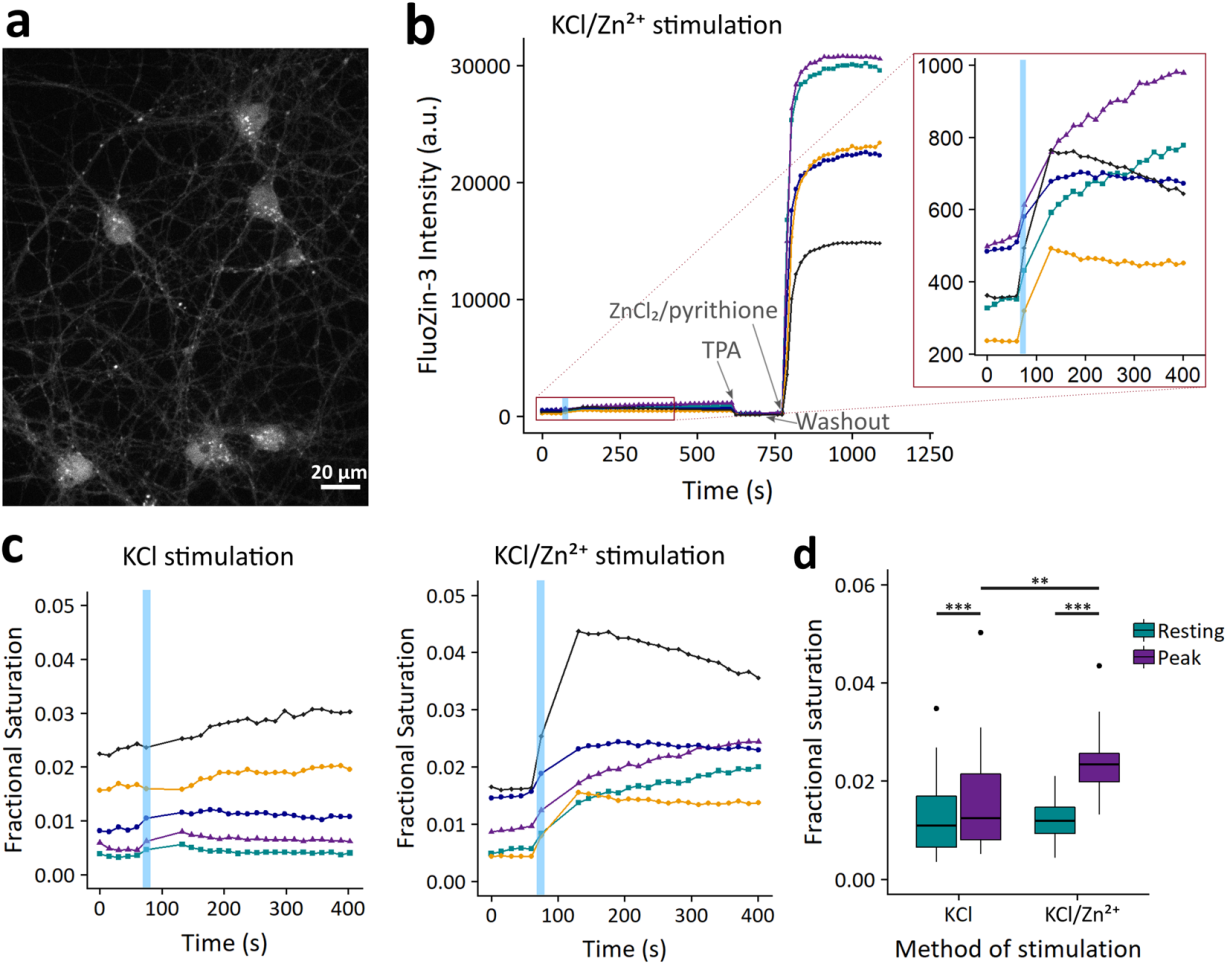
Intracellular Zn^2+^ signal in stimulated neurons. (**a**) Image of neurons treated with FluoZin-3 AM after stimulation with KCl in the presence of 10 µM ZnCl_2_. (**b**) Sample traces from a typical stimulation experiment. Each trace is derived from a region of interest within a different cell. 50 mM KCl stimulation was applied for 10 seconds (blue box) along with 10 µM ZnCl_2_, followed by a washout period and further imaging. Samples were calibrated by addition of 10 µM Zn^2+^ chelator TPA and then 10 µM ZnCl_2_/2.5 µM pyrithione in order to determine minimal and maximal signal for calculation of fractional saturation. Inset shows greater resolution for the time period around stimulation. Fluorescence intensity is background-corrected. (**c**) Sample traces from typical experiments described in (**b**), displayed as the calculated fractional saturation. Representative traces are shown for samples treated with KCl in the absence (left) or presence (right) of 10 μM extracellular ZnCl_2_. For clarity, only the first 400 seconds are shown. (**d**) Box plot of FluoZin-3 fractional saturation levels at rest and at peak level after KCl stimulation with or without 10 µM extracellular ZnCl_2_. n = 32 cells in 3 separate biological replicates for KCl stimulation alone and n = 18 cells in 3 separate biological replicates for the KCl/Zn^2+^ condition. Dots correspond to outliers, defined as points lying over (3^rd^ quartile + 1.5 × (interquartile range)). ***p < 0.001 or **p < 0.01 as assessed with a two-sided Wilcox Signed Rank test for paired data within conditions (KCl: test statistic V = 0, p = 4.6e-10; KCl/Zn^2+^: test statistic V = 0, p = 7.6e-09) and a two-sided Mann-Whitney U unpaired test between the KCl and KCl/Zn^2+^ resting and peak conditions (test statistic W = 131, p = 0.0012). No significant difference between resting conditions was observed (test statistic W = 276, p = 0.818).

Detecting Zn^2+^ in synaptic vesicles with fluorescent dyes is not straightforward. While some literature has described the intracellular Zn^2+^-specific dye FluoZin-3 AM as having vesicular localization^32,33^, we have previously shown that FluoZin-3 AM stains various cellular compartments including the cytosol, nucleus, Golgi apparatus, and lysosome^27,34^, and indeed in hippocampal neurons we see primarily a cytosolic and nuclear localization, with some aggregation in other subcellular compartments (see Fig. 4a for FluoZin-3 AM localization). Another reported vesicle-specific Zn^2+^ fluorescent dye, SpiroZin2, largely colocalizes with lysosomes^34,35^, and in our neuron cultures seems restricted to compartments in the cell body or rapidly trafficking along processes (Supplemental Movie 2). Thus, FluoZin-3 AM and SpiroZin2 were deemed unsuitable for synaptic vesicle Zn^2+^ detection in our system. We also attempted to use ZIMIR, a Zn^2+^-specific membrane dye, to quantify Zn^2+^ release upon stimulation (Supplemental Fig. S7), but were unable to show any stimulation-specific change in fluorescence.

Synaptic vesicle localization and dynamics have been previously visualized with stimulation-dependent uptake of membrane-bound FM dyes^36,37^. We adapted this technique to load synaptic vesicles with a membrane-impermeant version of FluoZin-3 (Fig. 3b). Briefly, we electrically stimulated cells in media containing both membrane dye FM 4–64 and a high concentration of FluoZin-3. As vesicles fuse with the plasma membrane fluorescent dyes can diffuse from the membrane or media into the vesicular lumen. We observed vesicular puncta that colocalized with FM 4–64 when this procedure was carried out with FluoZin-3 complexed with exogenous Zn^2+^ (Fig. 3b, FluoZin-3/Zn^2+^ loaded), suggesting the soluble FluoZin-3/Zn^2+^ complex can be taken up into vesicles upon neuron stimulation. However, in the absence of pre-loading with exogenous Zn^2+^, the FluoZin-3 signal was extremely dim and no clear vesicular puncta were visible (Fig. 3b, FluoZin-3 loaded). As this is an intensity-based dye, lack of signal could be due to several factors, including limited amount of dye uptake, slow dynamics of Zn^2+^ reloading after release into the extracellular space, or an increase in the *K*_*d*_ of FluoZin-3 for Zn^2+^ in the vesicular environment. We attempted to shift the equilibrium by adding extracellular Zn^2+^ after loading, but this had little effect on the vesicular FluoZin-3 signal (Supplemental Fig. S8). It is also possible that the amount of FluoZin-3 successfully loaded in vesicles was insufficient to compete away endogenous Zn^2+^ ions from the glutamate present in the vesicles, as glutamate likely coordinates Zn^2+^ to some extent^38,39^.

Cumulatively, these experiments suggest that while dissociated hippocampal neurons do correctly express and localize ZnT3, we were unable to confirm the presence of synaptic Zn^2+^. These results could indicate a lack of synaptic Zn^2+^, or they could reflect limitations of the applied tools in allowing us to detect it.

### Characterization of Zn^2+^ dynamics upon stimulation and global analysis of downstream differential gene expression

In order to visualize Zn^2+^ dynamics in excited neurons, we imaged neurons with cell-permeant Zn^2+^-specific dye FluoZin-3 AM during stimulation with KCl (Fig. 4). FluoZin-3 AM has been shown to be unresponsive to physiological perturbations of Ca^2+^ concentrations^40^, so changes in fluorescence during neuron stimulation specifically represent Zn^2+^ dynamics. We observed that with a 10 second KCl treatment alone, neurons exhibited a small but significant rise in cytosolic Zn^2+^, which often recovered to baseline values after the treatment was removed, although some cells had a more sustained response (Fig. 4c,d, KCl stimulation). We detected a similar Zn^2+^ rise using the ZapCV2 sensor (Supplemental Fig. S9). With FluoZin-3 AM, we observed that this Zn^2+^ signal increased if neurons were treated with KCl in the presence of 10 μM extracellular Zn^2+^, and the cells showed a similar variation in length of response (Fig. 4b,c, KCl/Zn^2+^ stimulation). Mild KCl treatment thus induces an endogenous rise in cytosolic Zn^2+^ that can be potentiated by extracellular Zn^2+^ addition (Fig. 4d). Using the *in vitro K*_*d*_ of FluoZin-3 (9.1 nM)^41^, the measured rise corresponds to an increase in Zn^2+^ concentration from roughly 110 pM to 150 pM in the absence of exogenous Zn^2+^, and from 110 pM to 220 pM in the presence of 10 µM exogenous Zn^2+^. The estimated concentrations indicate that neurons experience a modest increase in labile Zn^2+^ upon stimulation.

**Figure 5:**
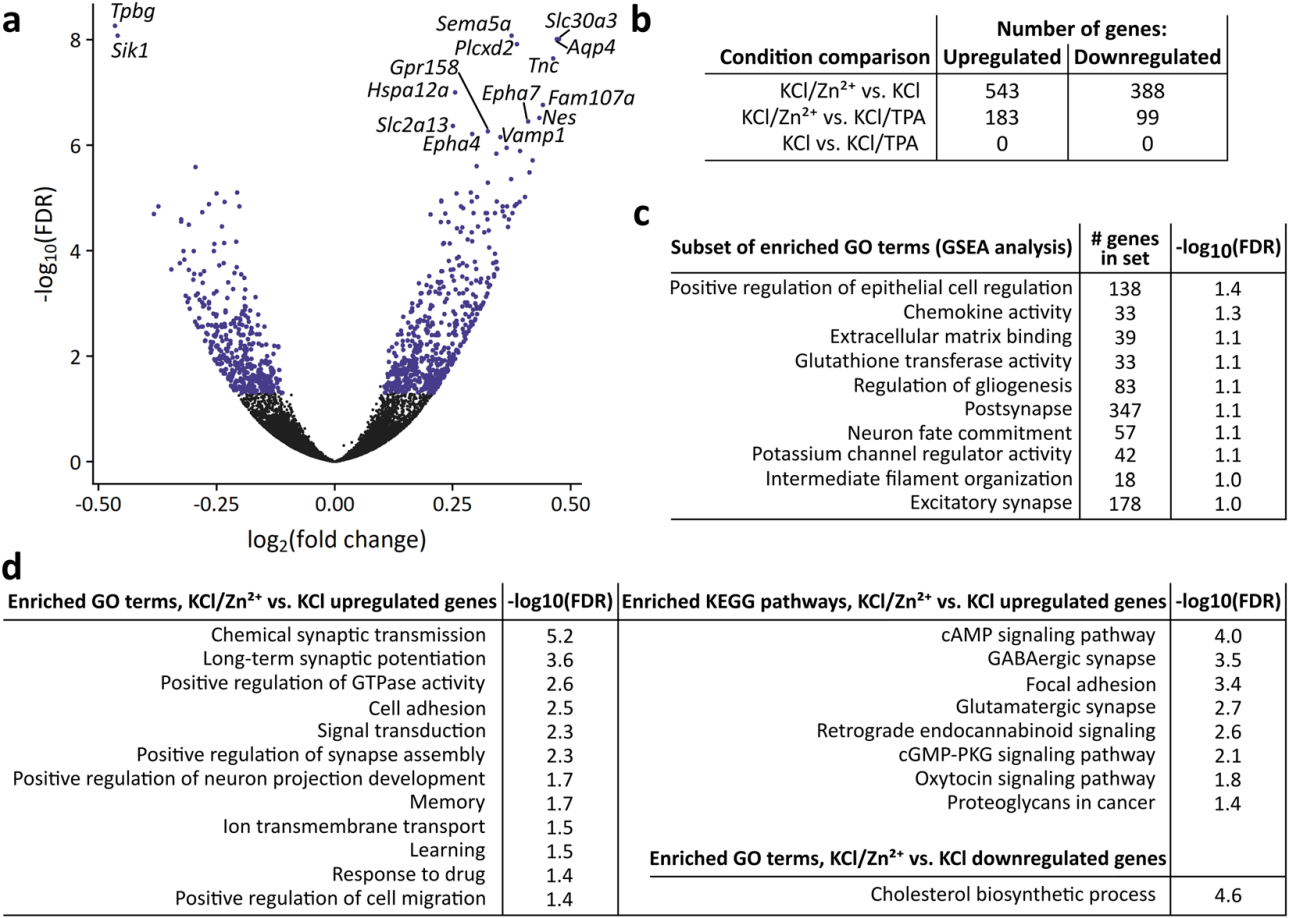
RNA-Seq of dissociated hippocampal neurons stimulated with and without Zn^2+^ or Zn^2+^-specific chelator TPA. (**a**) Volcano plot of gene expression for all genes between KCl/Zn^2+^ and KCl stimulation conditions, providing measures of how much gene expression changed and the confidence in calling that gene differentially expressed. Positive fold changes indicate that genes were upregulated in the KCl/Zn^2+^ condition. The top 13 upregulated and 2 downregulated genes have been labeled (all genes with FDR < 10^−6^). FDR = false discovery rate, a commonly used metric to assess significance among many hypothesis tests. Blue dots highlight all genes deemed significantly differentially expressed, with fold change magnitude >0.1 and FDR < 0.05. (**b**) Number of genes differentially expressed in pairwise comparisons of conditions. (**c**) Gene Set Enrichment Analysis (GSEA) results examining whether sets of genes associated with certain human gene ontology terms are enriched among genes upregulated in the KCl/Zn^2+^ condition as compared to the KCl condition. These gene sets are a relevant subset of 50 gene sets found to be enriched with an FDR < 0.1. (**d**) Analysis of enrichment of GO terms and KEGG pathways between KCl/Zn^2+^ and KCl conditions as assessed using the DAVIDtools online functional annotation tool. Enrichment is assessed as terms/pathways being proportionally more associated with the differentially expressed genes than with the transcriptome as a whole. All terms and pathways are listed that were found to be enriched in upregulated or downregulated genes with an FDR < 0.05. FDR = false discovery rate, GO = gene ontology, KEGG = Kyoto Encyclopedia of Genes and Genomes.

We broadly characterized the immediate transcriptional effects of the KCl-dependent Zn^2+^ increase by performing RNA-Seq on cells harvested 90 minutes after treatment with KCl in the presence and absence of extracellular Zn^2+^ or the membrane-permeable Zn^2+^-specific chelator TPA (Fig. 5). We used the same 10 second KCl stimulation as our imaging experiments in order to minimize cell stress and identify transcriptional effects arising from subtle Zn^2+^ increases that may more closely mimic physiological signals than extended treatment with high concentrations of Zn^2+^. It is well established that KCl treatment induces membrane depolarization and Ca^2+^ influx in excitable cells such as neurons. However, the KCl treatment remained constant across our conditions, and comparisons across conditions were thus representative specifically of Zn^2+^ perturbations. Differential expression analysis indicated that mild exogenous Zn^2+^ treatment during stimulation significantly alters expression of 931 genes, which can be seen as the blue dots in a volcano plot of the statistical significance vs. fold change of differentially expressed genes (Fig. 5a,b, Supplementary Dataset S1). In contrast, KCl stimulation in the presence of TPA had no detectable effect on gene expression compared to KCl treatment alone, likely indicating that TPA had little effect on Zn^2+^ dynamics during the short time (10 sec) of stimulation. Less overall differential expression was observed between the KCl/Zn^2+^ and KCl/TPA conditions than between the KCl/Zn^2+^ and KCl conditions. This observation could result from a high degree of variability among the biological replicates for KCl/TPA and intermediate values based on principal component analysis (PCA), which resulted in fewer genes identified as differentially expressed (Supplemental Fig. S10) We note that while the number of genes whose expression is altered by Zn^2+^ perturbations is exciting, the degree of change is small, likely because of the exceptionally mild Zn^2+^ perturbations and heterogeneity in both the Zn^2+^ signal (Fig. 4) and transcriptional response (PCA in Supplemental Fig. S10). We attempted to validate the changes in mRNA by RT-qPCR for three hits (ZnT3, Zip10, and Sik1) and found that we could reproduce the overall trend in expression for two of the three genes but the effects were not statistically significant due to small sample size (Supplemental Fig. S11).

We examined enrichment of gene ontology (GO) terms between KCl/Zn^2+^ and KCl stimulation conditions using both Gene Set Enrichment Analysis (GSEA) and the Functional Analysis tool in the DAVIDtools Suite (Fig. 5c,d). GSEA measures whether genes in a gene set (related to a certain GO term, in this case) are distributed at random in a dataset ranked by fold change between two conditions, or whether the set genes are over-represented in either the upregulated or downregulated subsets of the dataset. In contrast, the DAVIDtools Functional Analysis tool can determine whether a certain GO term is related to more genes that are significantly upregulated than it is to genes in the genome at large. Both of these methods indicated that a larger Zn^2+^ signal upregulates expression of genes important for synaptic structure and transmission, signal transduction, extracellular matrix regulation, and cytoskeletal rearrangements, which corroborates observations that Zn^2+^ plays a role in neuronal plasticity.

We noted that the most upregulated gene observed upon stimulation with KCl/Zn^2+^ was ZnT3 (*Slc30a3*, Fig. 5a, Supplemental Dataset S1), indicating that a larger Zn^2+^ signal induces neurons to shift Zn^2+^ homeostasis to expand their synaptic vesicle Zn^2+^ pool. *Slc39a10* (ZIP10), a plasma membrane-localized cytosolic Zn^2+^ importer, was also significantly upregulated, potentially allowing for more Zn^2+^ uptake from the extracellular space (Supplementary Dataset S1). In order to determine if other Zn^2+^-binding genes were altered, we performed GSEA with gene sets related to Zn^2+^ or Zn^2+^-binding, as derived from the Gene Ontology Consortium, the Protein Data Bank (PDB), or UniProt. None of these Zn^2+^-related gene lists were enriched between any of our conditions, suggesting that the expression of most genes encoding for Zn^2+^-binding proteins is not widely altered after a mild, transient Zn^2+^ signal.

We observed several other interesting transcriptional targets of Zn^2+^ signaling, including an upregulation in several members of the MAPK pathway (*Mapk1*, *Mapk4*, *Fgfr3*, *Frs2*, *Rapgef2*, *c-Jun*) and *Fgf13*, a non-secretory growth factor that has several roles in axonal outgrowth and excitatory neurotransmission. In addition, Zn^2+^ induced transcription of many subunits of glutamate and gamma-aminobutyric acid (GABA) receptors (*Gria1*, *Gria2*, *Gria3*, *Grin2A*, *Grik4*, *Grm7*, *Gabra1*, *Gabra3*, *Gabra5*, *Gabrb3*, *Gabrd*, *Gabre*), providing further support that the observed Zn^2+^ signal may contribute to synaptic growth, seemingly of both excitatory and inhibitory neurons.

The combination of imaging and sequencing data of dissociated hippocampal neurons following short KCl stimulation with or without extracellular Zn^2+^ indicates that a relatively small Zn^2+^ signal (~2-fold increase on the order of hundreds of pM) has significant and widespread effects on gene expression. Moreover, Zn^2+^-dependent responses are involved in many processes that are essential to neuronal plasticity.

## Discussion

In this work, we sought to characterize dissociated hippocampal cultures with respect to cytosolic and synaptic Zn^2+^, quantify Zn^2+^ dynamics upon stimulation, and carry out an unbiased screen of the global changes in gene expression that result from Zn^2+^ dynamics to identify possible molecular players that underlie Zn^2+^-dependent changes in neuronal functions and processes. In characterizing dissociated hippocampal neuron cultures with respect to synaptic Zn^2+^, we showed that neurons express and correctly localize the synaptic Zn^2+^ transporter ZnT3. However, despite using a variety of imaging tools, we were unable to determine whether the presence of this transporter actually promotes accumulation of Zn^2+^ in synaptic vesicles in dissociated neurons. These studies highlight the limitations of existing tools for defining synaptic Zn^2+^ in individual cells; the histological Timm stain did not provide sufficient contrast in isolated cells, small molecule membrane-permeable fluorescent dyes FluoZin-3 AM and SpiroZin-2 did not localize explicitly to synaptic vesicles, and extracellular probes FluoZin-3 and ZIMIR lacked sensitivity and did not yield reproducible results. These tool limitations do not provide definitive evidence that synaptic Zn^2+^ is absent from dissociated cultures. In contrast, using a different detection system, a recent study observed release of Zn^2+^ from stimulated cortical neurons and calculated the local synaptic Zn^2+^ concentration as approximately 100 nM^25^, which is in accordance with previous estimates^15,16,42^. Although we could not definitively confirm the presence of synaptic Zn^2+^, we were able to rigorously define the resting cytosolic labile Zn^2+^ concentration to be approximately 60 pM (using ZapCV2) to 110 pM (using FluoZin3 AM), and we observed a small transient rise in Zn^2+^ to roughly 150 pM upon depolarization with KCl. This transient rise in cytosolic Zn^2+^ was potentiated to approximately 220 pM when neurons were depolarized in the presence of 10 µM Zn^2+^.

As the importance of Zn^2+^ in neurons has become more widely recognized, an increasing number of studies have focused on downstream effects of Zn^2+^ signaling, including the enhancement of long term potentiation (LTP)^19^. Several studies, however, have induced signaling changes with intensive exogenous Zn^2+^ conditions, such as 50–300 µM Zn^2+^ for time periods as long as 1 hour^43–45^. These conditions are likely to produce intracellular Zn^2+^ concentrations much higher than observed physiologically *in vivo*, and have been shown to cause substantial cell stress and cell death^46–48^.

We performed global mRNA sequencing on neurons under conditions that produced cytosolic Zn^2+^ signals that were in the sub-nanomolar range. Importantly, our results did not show an upregulation of stress-response genes, suggesting that this mild Zn^2+^ increase does not induce neuronal stress. We did observe that a subtle Zn^2+^ signal induced a significant upregulation of genes related to neurite growth and synaptic function, including many neurotransmitter receptor subunits and the Zn^2+^ transporter ZnT3. Interestingly, while Zn^2+^ has been well documented to alter the immediate responses of glutamate and GABA receptors^2,15,49,50^ and has been implicated in modulating LTP in a number of electrophysiology experiments^1,2,15,19,20^, to our knowledge no studies have documented a transcriptional component of Zn^2+^-dependent synaptic regulation. It is unclear to what extent the induced intracellular Zn^2+^ signal reflects *in vivo* neuronal Zn^2+^ signals; however, our results suggest that sub-nanomolar increases in cytosolic Zn^2+^ have the potential to alter the expression of genes that have been implicated in late-phase LTP.

Another intriguing gene that was upregulated by Zn^2+^ in our neuron cultures encodes the protein FGF13, a growth factor that is highly expressed in developing cortical and hippocampal neurons. FGF13 has two main known functions. The first is that FGF13 polymerizes and stabilizes microtubules at axon growth cones^51–53^, and in this capacity FGF13 upregulation contributes to the observed Zn^2+^-dependent neurite growth induction. The second is that FGF13 limits the membrane presentation of voltage-gated sodium channels in dendritic spines, and knockout of the *Fgf13* gene leads to greater excitatory potentials and epilepsy^54,55^. ZnT3 knockout mice, which lack synaptic Zn^2+^, are also prone to acute seizures^56,57^, and while there are many potential links between Zn^2+^ and epilepsy^58^, FGF13 may provide an example of how Zn^2+^-dependent transcription could modulate neuron excitability.

Our results indicate that a transient Zn^2+^ signal has transcriptional effects in neurons. One signaling pathway likely to be involved is the MAPK pathway, which has previously been shown to be activated by Zn^2+^ ^12,13,44^. We found that key components of the pathway (*Mapk1*, *Mapk4*) were upregulated upon increased cytosolic Zn^2+^, along with other associated factors (*Epha4*, *Ephb1*, *Fgfr3*, *Rapgef2*) that have been shown to play important roles in neurite growth, branching, and guidance^59–62^. Additionally, the downstream transcription factor c-Jun (*Jun*) was significantly upregulated, although we saw no significant difference in expression of any of its binding partners (*Fos*, *FosB*, *Fosl1*, *Fosl2*, *Junb*, *Jund*)^63^. c-Jun has been implicated in signaling for axon regeneration and neuron apoptosis, but we did not observe any significant difference in expression among any of the genes normally coexpressed with *Jun* in either phenotype (*Mapk8*, *Mapk9*, *Mapk10*, *Gap43*, *Atf2*, *Atf3*, *Smad1*)^64–67^. Thus, although it seems likely that Zn^2+^ activates the MAPK pathway, which may then affect Zn^2+^-dependent transcription through c-Jun, the mechanisms underlying these steps still require further study.

In summary, our global RNA-Seq results suggest that sub-nanomolar Zn^2+^ signals are sufficient to significantly alter gene expression in a relevant model system. Moreover, in neurons these gene expression changes correlate to physiological changes that are consistent with previous observations of the effect of Zn^2+^ on neuronal signaling.

## Materials and Methods

### Neuron isolation/culture

Dishes/slides were coated overnight with 1 mg/mL Poly-D-lysine hydrobromide in 15 mM sodium borate. Glass slides for imaging were washed thoroughly and coated in 50 µM iMatrix-511 (Clontech) until neuron plating.

All animal work was approved by the Institutional Animal Care and Use Committee of CU Boulder, protocols #1407.01 and #2547 and performed in accordance with the relevant guidelines and regulations. E18 rat hippocampi were either isolated from animals or ordered from BrainBits, LLC. For animal isolation, timed pregnant Sprague Dawley rats were obtained from Charles River Laboratories. Pregnant rats were sacrificed with carbon dioxide and fetal brains were micro-dissected to isolate hippocampi. Pooled hippocampi were washed with digestion medium (1X HBSS, 10 mM HEPES, 5 µg/mL gentamicin, pH 7.2) and digested 30 min in digestion medium containing 20 U/mL papain (Worthington). Samples were then washed with plating medium (MEM, 5% FBS, 0.6% wt/vol glucose) and dissociated by sequentially passing 5–10 times through full-diameter, then half-diameter flame-polished Pasteur pipets. Cells were plated on treated slides at a density of 20,000 cells/cm^2^ for imaging. Cells intended for RNA isolation were plated on treated dishes at a density of 60,000 cells/cm^2^. Cells were fed 3–4 hours after plating with glial-conditioned neuron culture medium (Neurobasal Medium, 2% B27 supplement, 0.3x GlutaMAX supplement, all obtained from Thermo Fisher), and ½ media was replaced on day *in vitro* (DIV) 3, DIV 6, and DIV 13. Cultures were treated with 4 µM cytosine arabinoside from DIV 3 to DIV 6 to restrict mitotic cell proliferation. Cultures were grown in a cell culture incubator at 37 °C and 5% CO_2_.

Glial cells were isolated from neonatal cortical tissue collected from neonatal rats sacrificed by decapitation with sharp scissors. Cells were dissociated as with hippocampal samples, then plated on standard cell culture dishes. Cells were fed initially and every 3–4 days with glial medium (DMEM, 5% FBS, 0.5% pen/strep) until confluent. To generate glial-conditioned medium, neuron culture medium was added to confluent glial cultures for one day, then filtered through a 0.20 µm filter prior to addition to neurons.

### Materials

Genetically encoded Zn^2+^ Förster Resonance Energy Transfer (FRET) sensor NES-ZapCV2 was used for all resting cytosolic Zn^2+^ measurements (see RRID:Addgene_112060^68^). Sensor was either transfected 2 days prior to imaging with Lipofectamine 3000 (Thermo Fisher) according to the manufacturer’s protocol, or nucleofected prior to neuron plating with a Lonza Nucleofector 2b instrument using the Rat Neuron Nucleofector Kit (Lonza) protocol. Nucleofected sensor remained fluorescent in cultures through DIV 12–13.

The following fluorescent small molecule dyes were obtained from Thermo Fisher: FluoZin-3, FluoZin-3 AM, and FM 4–64. Stock solutions of FluoZin-3 and FluoZin-3 AM were prepared at 1 mM in DMSO. Stock solutions of FM 4–64 were prepared at 5 mg/mL in water. The small molecule dye ZIMIR was obtained from VitalQuan, and stock solutions were prepared at 1 mM in DMSO.

The Zn^2+^-specific chelator tris(2-pyridylmethyl)amine (TPA), ZnCl_2_, chelex, and the ionophore 2-mercaptopyridine *N*-oxide (pyrithione) were purchased from Sigma-Aldrich. TPA stock solutions were prepared at 20 mM in DMSO. ZnCl_2_ stock solutions were prepared at 1 mg/mL in water treated overnight with chelex. Pyrithione stock solutions were prepared at 5 mM in DMSO.

For synaptic vesicle dye loading experiments, glutamate receptor antagonists 6-Cyano-7-nitroquinoxaline-2,3-dione (CNQX) and D-(−)-2-Amino-5-phosphonopentanoic acid (D-AP5) were purchased from Abcam. CNQX stock solutions were prepared at 5 mM in DMSO, while D-AP5 stock solutions were prepared at 50 mM in water. For dye quenching, advasep-7 (Biotium) was dissolved in water at a stock concentration of 100 mM.

Primary antibodies and peptide controls were purchased from Synaptic Systems: anti-ZnT3 (RRID:AB_2189665), anti-Homer1 (RRID:AB_887730), anti-Synapsin (RRID:AB_1106784), ZnT3 peptide (#197-0P). Secondary antibodies were obtained from Thermo Fisher: anti-mouse AF488 (RRID:AB_2534088), anti-guinea pig AF488 (RRID:AB_2534117), anti-rabbit AF568 (RRID:AB_2534017), anti-mouse AF594 (RRID:AB_2534091).

Resting neuron imaging media (RNIM) was formulated as follows: 145 mM NaCl, 3 mM KCl, 1.5 mM CaCl_2_, 1 mM MgCl_2_, 10 mM HEPES, 10 mM glucose, pH 7.4. High-potassium neuron imaging media (KNIM) was made as a 2X K^+^ solution (51 mM NaCl, 97 mM KCl, 1.5 mM CaCl_2_, 1 mM MgCl_2_, 10 mM HEPES, 10 mM glucose, pH 7.4), which when added 1:1 to RNIM gave concentrations of 98 mM NaCl and 50 mM KCl. All media was prepared in chelex-treated water.

### Equipment

Samples for all imaging experiments were imaged on a Nikon Ti-E spinning disc confocal microscope equipped with Nikon Elements software, Ti-E perfect focus system, Yokogawa CSU-X1 spinning disc head, Andor 888 Ultra EMCCD camera and Oko Labs enclosed environmental chamber set at 37 °C.

Samples for each type of imaging experiment except immunofluorescence experiments were also imaged on a Nikon Ti-E widefield microscope equipped with Nikon Elements software, Ti-E perfect focus system, Andor iXon3 EMCCD camera, Sutter Instruments LD-LS/30 xenon arc lamp, and Sutter Instruments Lambda 10-3 filter changer.

For electrical stimulation, an IonOptix Myopacer cell stimulator was equipped with a custom set of platinum wire electrodes. Stimulations were performed with 30 V bipolar waveform 10 ms pulses at 5 Hz for a duration of 1 minute, unless specified otherwise.

RNA extraction for next-generation sequencing was performed using a Promega Maxwell RSC Instrument.

Quantitative PCR was performed using a BIO-RAD CFX384 Real Time PCR Detection System instrument.

### *In vitro* Kd determination

Protein purification, *in vitro* fluorescence measurements at different Zn^2+^ concentrations, and fitting to determine apparent sensor *K*_*d*_ were done exactly as described previously^26^, with the exception that emission fluorescence intensities at 475 nm were used for λ_1_ values rather than intensities at 481 nm. Fits were determined based on data from 11 titration experiments.

### Intracellular FRET sensor calibrations

Measurements were taken on the Nikon spinning disc confocal microscope using CFP (445 nm excitation, 482/35 nm emission) and FRET (445 nm excitation, 540/30 nm emission) channels, acquiring images with a 40X (NA 0.95) air objective at 300 ms exposure, EM multiplier 300, 10 MHz camera readout speed, 20% laser power, and binning pixels 2 × 2. Measurements were also taken on the Nikon widefield microscope using CFP (434/16 nm excitation and 470/24 nm emission) and FRET (434/16 nm excitation and 535/20 nm emission) channels, acquiring images with a 20X (NA 0.75) or 40X (NA 0.95) air objective at 4–600 ms exposure, EM multiplier 300, 1 MHz camera readout speed, with a neutral density filter (ND8) restricting lamp light to 12.5% maximum.

Neuron cultures (DIV 10–14) were washed and put in RNIM at least ten minutes prior to imaging. Baseline measurements were obtained for 5–15 minutes. Calibrations were performed by adding 10 µM TPA for 2–5 minutes, followed by wash out with RNIM and addition of 10 µM ZnCl_2_/0.5–2 µM pyrithione. Measurements were taken for several minutes after a maximum FRET ratio was reached. For KCl stimulation experiments, KNIM was mixed 1:1 with RNIM and measurements were taken for 400–600 seconds before washing out with RNIM and performing a calibration as above.

### Synaptic vesicle dye loading

Cultures were imaged on the Nikon spinning disc confocal microscope using GFP (FluoZin-3: 488 nm excitation, 525/50 nm emission) and GFP/RFP (FM 4–64: 488 nm excitation, 620/60 nm emission) channels, acquiring images with a 100X (NA 1.45) oil objective at 300 ms exposure, EM multiplier 300, 10 MHz camera readout speed, and 15–20% laser power. Measurements were also taken on the Nikon widefield microscope using GFP (FluoZin-3: 480/20 nm excitation and 500/20 nm emission) and GFP/RFP (FM 4–64: 480/20 nm excitation and 610/50 nm emission) channels, acquiring images with a 60X (NA 1.40) oil objective at 300 ms exposure, EM multiplier 300, 1 MHz camera readout speed, with a neutral density filter (ND4) restricting lamp light to 25% maximum.

Neuron cultures (DIV 10–14) were incubated for 2 minutes in RNIM containing 5 µg/mL FM 4–64, 5–50 µM FluoZin-3, 10 µM CNQX, and 100 µM D-AP5. Without changing media, cultures were electrically stimulated for 1 minute, then incubated 5 minutes to accommodate compensatory endocytosis. Cells were washed twice and incubated in RNIM containing 1 mM advasep-7, 10 µM CNQX, and 100 µM D-AP5 to quench extracellular dye.

RNIM containing 50 µM ZnCl_2_ was added to some cultures to attempt to visualize vesicles. Cultures were also further stimulated to visualize dye exocytosis with electrical stimulation, KNIM, or RNIM containing 50 µM glutamate. For positive controls, 10 µM ZnCl_2_ was included in the initial loading solution to pre-load FluoZin-3 with Zn^2+^ before endocytosis.

### Imaging Zn^2+^ release with ZIMIR

Cultures were imaged on the Nikon spinning disc confocal microscope using a GFP channel (488 nm excitation, 525/50 nm emission), acquiring images with a 100X (NA 1.45) oil objective at 200 ms exposure, EM multiplier 300, 10 MHz camera readout speed, and 20% laser power. Images were also acquired with a 40X (NA 0.95) air objective at 300 ms exposure, EM multiplier 300, 10 MHz camera readout speed, and 20% laser power. Some measurements were taken on the Nikon widefield microscope using a GFP channel (480/20 nm excitation and 500/20 nm emission), acquiring images with a 40X (NA 0.95) air objective at 200 ms exposure, EM multiplier 300, and a 1 MHz camera readout speed, or with a 60X (NA 1.40) oil objective at 200 ms exposure, EM multiplier 300, 1 MHz camera readout speed, with a neutral density filter (ND4) restricting lamp light to 25% maximum.

Neuron cultures (DIV 10–14) were incubated for 20–30 minutes in 5 µM ZIMIR, then washed and imaged immediately. Electrical stimulation was applied, with stimulation protocols varied. The two most commonly used protocols were 30 V bipolar waveform 10 ms pulses at 10 Hz for a duration of 1–2 seconds, or 40 V bipolar waveform 10 ms pulses at 20 Hz for a duration of 10 seconds. Dye responsiveness was assessed by adding 10 µM TPA for 2–5 minutes, then washing out with RNIM and adding 10 µM ZnCl_2_. In some cases, 10 µM ZnCl_2_ was added to normal culture media for 0.5–48 hours prior to imaging, then washed out before dye incubation.

### Intracellular stimulation-induced Zn^2+^ measurements with FluoZin-3 AM

Measurements were taken on the Nikon spinning disc microscope using a GFP channel (488 nm excitation, 525/50 nm emission), acquiring images with a 40X (NA 0.95) air objective at 300 ms exposure, EM multiplier 300, 10 MHz camera readout speed, and 15% laser power.

Neuron cultures (DIV 10–14) were washed and incubated at room temperature in RNIM containing 5 µM FluoZin-3 AM for 30 minutes. Samples were washed in RNIM. Baseline measurements were obtained for 1 minute. Cells were then stimulated with a 10 second treatment of high K^+^ by mixing KNIM 1:1 with the RNIM already present. After 10 seconds, cultures were washed 3x with RNIM and measurements taken for 15 minutes. Calibrations were performed by adding 10 µM TPA for 2 minutes, then washing out with RNIM and adding 10 µM ZnCl_2_/0.5 µM pyrithione. Measurements were taken until several minutes after a maximum signal was observed.

### Image analysis

FRET sensor, ZIMIR, and FluoZin-3 AM imaging experiments were analyzed with a custom MATLAB script that imports ND2 experiment files, extracts metadata, registers images, allows for manual background and cell ROI selection, and generates raw and background-subtracted average intensity measurements. FRET ratios were calculated as background-subtracted (FRET fluorescence)/(CFP fluorescence). FRET and FluoZin-3 AM calibration data were manually inspected to obtain minimum and maximum values for FRET ratios or intensity measurements, which were used to calculate fractional saturation according to the formula:

$$FS=\frac{X-{F}_{min}}{{F}_{max}-{F}_{min}}$$

where X is the basal or peak measurement and F_min_ and F_max_ are the minimum and maximum values of either the FRET ratio (for FRET measurements) or background-subtracted fluorescence intensity (for FluoZin-3 AM measurements). Fractional saturation was converted to an approximate intracellular Zn^2+^ concentration according to the formula:

$$[Z{n}^{2+}]=\frac{{K}_{d}}{{(\frac{1}{FS}-1)}^{\frac{1}{n}}}$$

where K_d_ is the sensor dissociation constant, FS is fractional saturation as defined above, and n is the Hill coefficient (assumed to be 1 for FluoZin-3 AM).

Immunofluorescence (IF) images were decomposed by channel. Using MATLAB, images of multi-channel fluorescent beads or IF images of Homer1 and ZnT3 (594 nm secondary antibody) at DIV 6 were registered to correct for spectral anomalies between channels, then resulting registration parameters were applied to all IF images by channel. Integrated intensities were calculated on raw fluorescence of registered images, or on images subjected to adaptive thresholding according to the built-in MATLAB function adaptthresh (sensitivity = 0.25). Two-dimensional correlation coefficients were calculated on raw fluorescence intensities of registered images using the built-in MATLAB function corr2.

### RT-qPCR

For ZnT3 analysis over time, neurons at DIV 0 (extraction day) (1 replicate), DIV 1 (1 replicate), DIV7 (2 replicates), DIV 10 (2 replicates), DIV 14 (2 replicates), DIV 17 (1 replicate), and DIV 21 (1 replicate) were lysed and RNA extracted according to the Qiagen RNeasy kit protocol (Qiagen). RNA was reverse transcribed according to Protoscript II Reverse Transcriptase protocol (New England BioLabs), and quantitative PCR was performed with the TaqMan Fast Advanced Master Mix and gene-specific TaqMan assays (Applied Biosystems) according to the standard master mix protocol. TaqMan assays analyzed were Rn01472608_m1 (ZnT3), Rn01462662_g1 (GAPDH), and Rn00560865_m1 (Beta-2-microglobulin). 3–4 technical replicates were performed per sample. PCR cycle conditions were as follows: 50 °C, 2 min; 95 °C, 2 min; (95 °C, 3 s; 60 °C, 30 s) ×40 cycles.

For RNA-Seq validation analysis, neurons at DIV 14 were incubated in a 1:1 solution of RNIM/KNIM for 10 seconds, with and without the addition of 10 µM ZnCl_2_. Each of the two conditions was performed in triplicate. After treatment, samples were placed back in the neuron culture media and incubated for 90 minutes at 37 °C and 5% CO_2_. RNA extraction and reverse transcription were performed as in the previous paragraph. Quantitative PCR was performed with the TaqMan Fast Advanced Master Mix and gene-specific TaqMan assays (Applied Biosystems) according to the standard master mix protocol. TaqMan assays analyzed were Rn01472608_m1 (ZnT3), Rn01771489_m1 (ZIP10), Rn01429325_m1 (Sik1), Rn01462662_g1 (GAPDH), and Rn00560865_m1 (Beta-2-microglobulin). 3–4 technical replicates were performed per sample. Cycle conditions were as in the previous paragraph.

### Immunofluorescence (IF)

Cultures were prepared for IF at DIV 1, 7, 10, 14, 17, and 21. Samples were fixed for 10 minutes in PBS/3.7% paraformaldehyde, then quenched in PBS/20 mM NH_4_Cl for 5 minutes. Samples were blocked in PBS/10% goat serum for 30 minutes. Antibodies were diluted in PBS/2% goat serum. Samples were incubated with primary antibodies overnight and secondary antibodies for 30 minutes, with 4 × 10-minute washes between incubations. Samples were imaged on the Nikon spinning disc confocal microscope using a 100X (NA 1.45) oil objective.

Anti-ZnT3 was used as a 1:100 dilution of 1 mg/mL stock solution, and anti-Homer1 and anti-Synapsin were used as 1:200 dilutions of 1 mg/mL stock solutions. For ZnT3 peptide controls, 2.5 µg ZnT3 antibody was pre-incubated with 6 µg ZnT3 control peptide for 20 minutes prior to sample incubation. Secondary antibodies were used as 1:1000 dilutions of 2 mg/mL stock solutions.

### Timm’s stain

Neuron cultures were stained at DIV 10–14. Subsets of samples were treated prior to staining with 20–40 µM ZnCl_2_ for 20 minutes, or with 20 µM ZnCl_2_/2.5 µM pyrithione for 8 minutes. Samples were washed and incubated for 2 minutes in sulfide solution (0.027% Na_2_S dissolved in 82 mM Tris, pH 8), then washed and fixed for 10 min in PBS/3.7% paraformaldehyde and quenched for 5 min with PBS/20 mM NH_4_Cl. Samples were developed for 1 hour in developing solution prior to imaging. Developing solution was formulated by mixing aqueous solutions A (5.7 mM AgNO_3_), B (2% citric acid, 0.8% hydroquinone), and C (10.7% gum arabic) in the volume ratio 65:50:7 (A:B:C).

### Sequencing and computational pipeline

Neuron cultures (DIV 14) were placed at room temperature. Media was removed and neurons incubated in a 1:1 solution of RNIM/KNIM for 10 seconds, with and without the addition of 10 µM ZnCl_2_ or 10 µM TPA. Each of the three conditions (Stimulation, stimulation + ZnCl_2_, stimulation + TPA) was performed in triplicate. After treatment, samples were placed back in the removed neuron culture media and incubated for 90 minutes at 37 °C and 5% CO_2_. Cells were lysed and RNA extracted according to the Promega Maxwell RSC simplyRNA isolation kit. Libraries were generated according to the Bioo NextFlex Dir RNA kit and sequenced on an Illumina NextSeq 500 using a high-output 2 × 75 paired-end protocol.

Rat reference genome files (rn6), including genome fasta file and gtf gene annotation file, were downloaded from Illumina iGenomes (https://support.illumina.com/sequencing/sequencing_software/igenome.html) on 7/31/2017.

Quality control of de-multiplexed fastq files was performed using fastQC (v0.11.2, https://www.bioinformatics.babraham.ac.uk/projects/fastqc/). The first ten bases of all reads were trimmed from each separate fastq file in a single-end fashion with trimmomatic (v0.32)^69^ and resulting fastq files were mapped in a paired-end fashion with tophat2 (v2.0.6, bowtie2 v2.0.2)^70^ using parameters –b2-very-sensitive -r 110–mate-std-dev 180 and including the rn6 genes GTF file. Resultant BAM files were indexed with samtools (v0.1.18)^71^.

The featureCounts function within the R package Rsubread (v1.24.2, R v3.3.0)^72^ was used to generate gene-level read counts, with parameters GTF.featureType = “exon”, GTP.attrType = “gene_id”, useMetaFeatures = TRUE, allowMultiOverlap = FALSE, largestOverlap = FALSE, strandSpecific = 2, isPairedEnd = TRUE, requireBothEndsMapped = TRUE, checkFragLength = TRUE, minFragLength = 50, maxFragLength = 60000, countChimericFragments = FALSE. Differential expression analysis was performed pairwise between conditions with the R package DESeq 2 (v1.14.1, R v3.3.0)^73^. Differentially expressed genes were filtered according to a false discovery rate (p_adj_) < 0.05, split into upregulated and downregulated genes, and analyzed with the DAVIDtools Functional Annotation Tool using default parameters (v6.8, https://david.ncifcrf.gov/)^74,75^.

For GSEA analysis, gene level counts were normalized to transcripts per million (TPM). GSEA (v3.0, http://software.broadinstitute.org/gsea/index.jsp)^76,77^ was then run on the TPM file with default parameters except for the following: Collapse dataset to gene symbols = false, Permutation type = gene_set, max set size = 3000, min set size = 5. Gene sets were downloaded from the Broad Institute Molecular Signatures Database (v6.2, http://software.broadinstitute.org/gsea/msigdb/index.jsp), collection C5 (GO gene sets). Gene sets for Zn^2+^-related genes were manually curated from mouse species-filtered searches of (1) the Gene Ontology Consortium (http://www.geneontology.org/) for genes annotated with the terms “zinc ion transport”, “cellular zinc ion homeostasis”, “response to zinc ion”, “zinc ion transmembrane transport”, or “zinc ion binding”, (2) the Protein Data Bank (https://www.rcsb.org/) for all structures containing ZN, and (3) UniProt (https://www.uniprot.org/) for “zinc”.

Principal component analysis was performed on samples based on gene-level TPM values with the built-in R function prcomp (R v.3.5.1).

### Statistical analysis and plotting

All statistical tests were performed in R (v3.5.1), and are detailed in individual figure legends. For comparison of Zn^2+^ responses to stimulation, two-sided Wilcox Signed Rank tests and two-sided Mann-Whitney U tests were used due to non-normality of original data (as assessed by a Shapiro Wilk test). Statistical tests assessing significance of differential expression were performed by the DESeq2 package, which accounts for multiple hypothesis testing corrections. Statistical tests assessing significance of GO term enrichment results were performed by the GSEA and DAVIDtools algorithms and accounted for multiple hypothesis testing corrections.

All fits and plots were generated with R (v3.5.1), using the packages ggplot2 (v3.0.0), ggrepel (v0.8.0), reshape2 (v1.4.3), extrafont (v0.17), and cowplot (v0.9.3).

## Supplementary information

Supplemental Movie 1

Supplemental Movie 2

Supplementary Information

Supplementary Dataset S1

## Data Availability

All raw next-generation sequencing data files and processed data files used to draw conclusions are available at the Gene Expression Omnibus, data series GSE126841.
