# Supplementary material for: Intracellular Zn2+ transients modulate global gene expression in dissociated rat hippocampal neurons
Source: Sci Rep. 2019 Jun 28;9:9411. doi: 10.1038/s41598-019-45844-2 (PMC6598991; doi:10.1038/s41598-019-45844-2)
Supplement: Supplementary file 3 — Supplementary Information [file 41598_2019_45844_MOESM3_ESM.pdf]

Intracellular Zn^{2+} transients modulate global gene expression in dissociated rat hippocampal neurons

Lynn Sanford, Margaret C. Carpenter, Amy E. Palmer*

Supplemental Figure S1: Representative images of cells expressing Zn^{2+} sensor ZapCV2

Supplemental Figure S2: *In vitro* binding curve for genetically encoded Zn^{2+} sensor ZapCV2

Supplemental Figure S3: RNA expression of Zn^{2+} transporter ZnT3 in cultured neurons

Supplemental Figure S4: Quantification of immunofluorescence signal

Supplemental Figure S5: Pre-synaptic localization of ZnT3

Supplemental Figure S6: Specificity of ZnT3 immunofluorescence in DIV 10 neuron cultures

Supplemental Figure S7**:** Fluorescence signal of the extracellular Zn^{2+}-specific membrane dye ZIMIR

Supplemental Figure S8: Zn^{2+} treatment of FluoZin-3 loaded neurons

Supplemental Figure S9: Zn^{2+} FRET sensor experiments in KCl-stimulated neurons

Supplemental Figure S10: Comparison of all replicates and treatment conditions in RNA-Seq data

Supplemental Figure S11: RT-qPCR of select hits from RNA-Seq experiment

Supplemental References

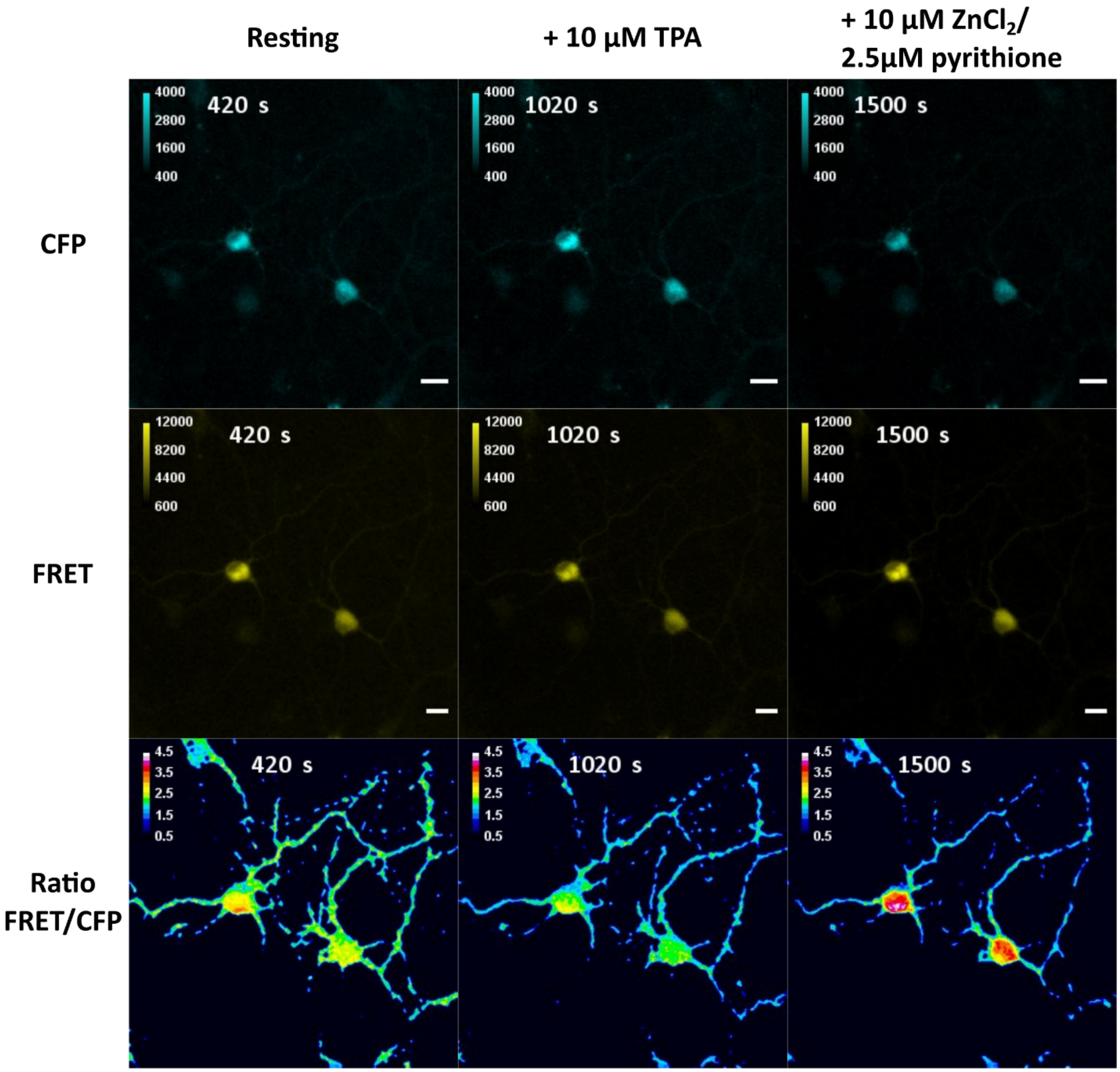

Supplemental Figure S1: Representative images of cells expressing Zn^{2+} sensor ZapCV2. CFP and FRET channels are shown with corresponding pixel intensity scales, along with ratio images of relevant neuronal areas within the field of view (ratio images are repeated from Fig. 1a). Resting images were taken before exogenous Zn^{2+} perturbation, 10 µM TPA treatment shows unbound sensor (higher CFP intensity, lower FRET intensity, lower ratio), and 10 µM $ZnCl_2$/2.5 µM pyrithione treatment shows fully bound sensor (lower CFP intensity, higher FRET intensity, higher ratio). Scale bars = 20 µm.

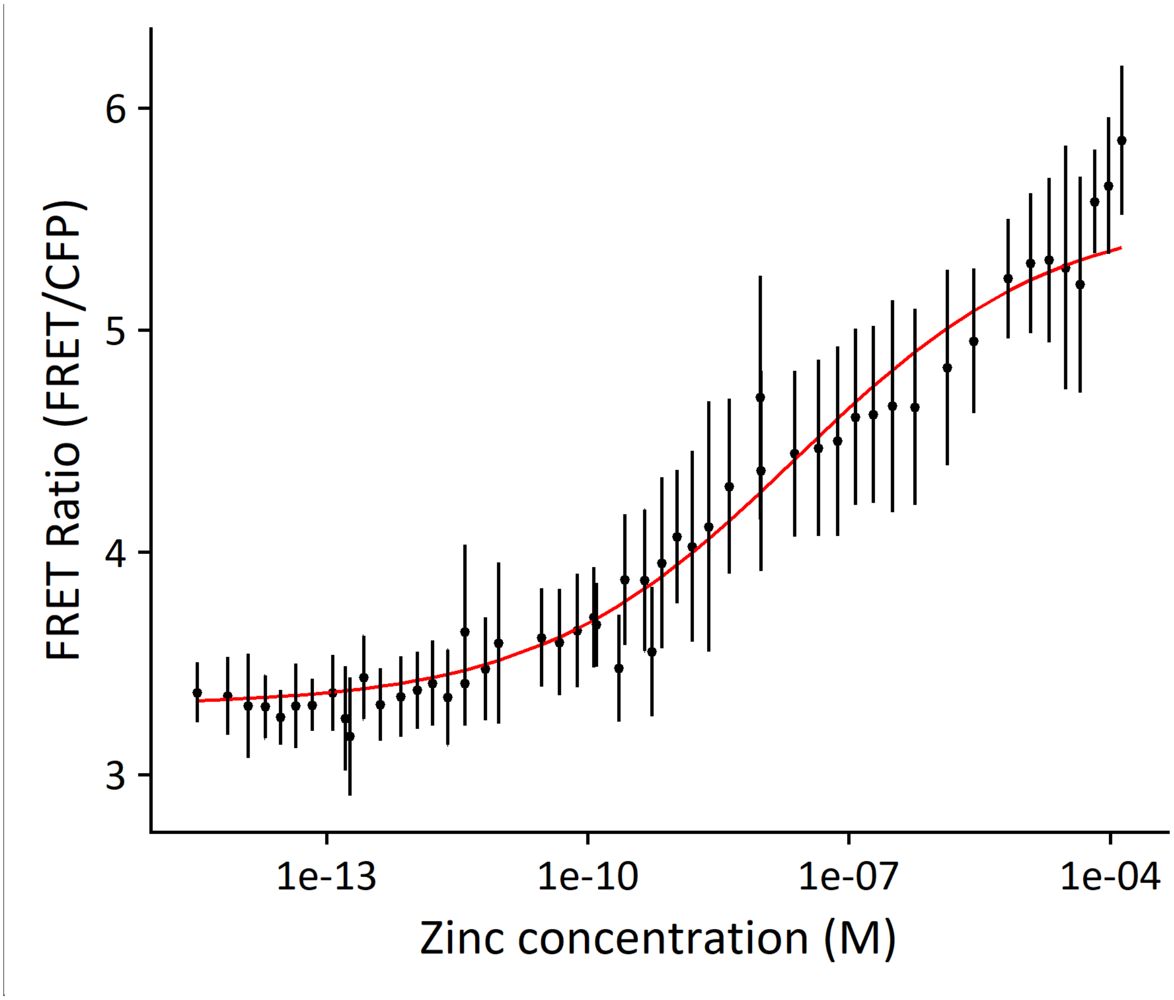

Supplemental Figure S2: *In vitro* binding curve for genetically encoded Zn^{2+} sensor ZapCV2. Sensor protein was expressed and purified from *E. coli*, and sensor fluorescence (Excitation: 420 nm, donor emission: 475 nm, acceptor emission: 529 nm) was measured in different buffered Zn^{2+} concentrations. The binding curve shown yielded the parameters K_d = 5.3 nM ± 1.1 nM and Hill coefficient n = 0.29 ± 0.02. Error bars indicate the standard deviation of measurements, and parameter error values correspond to standard error.

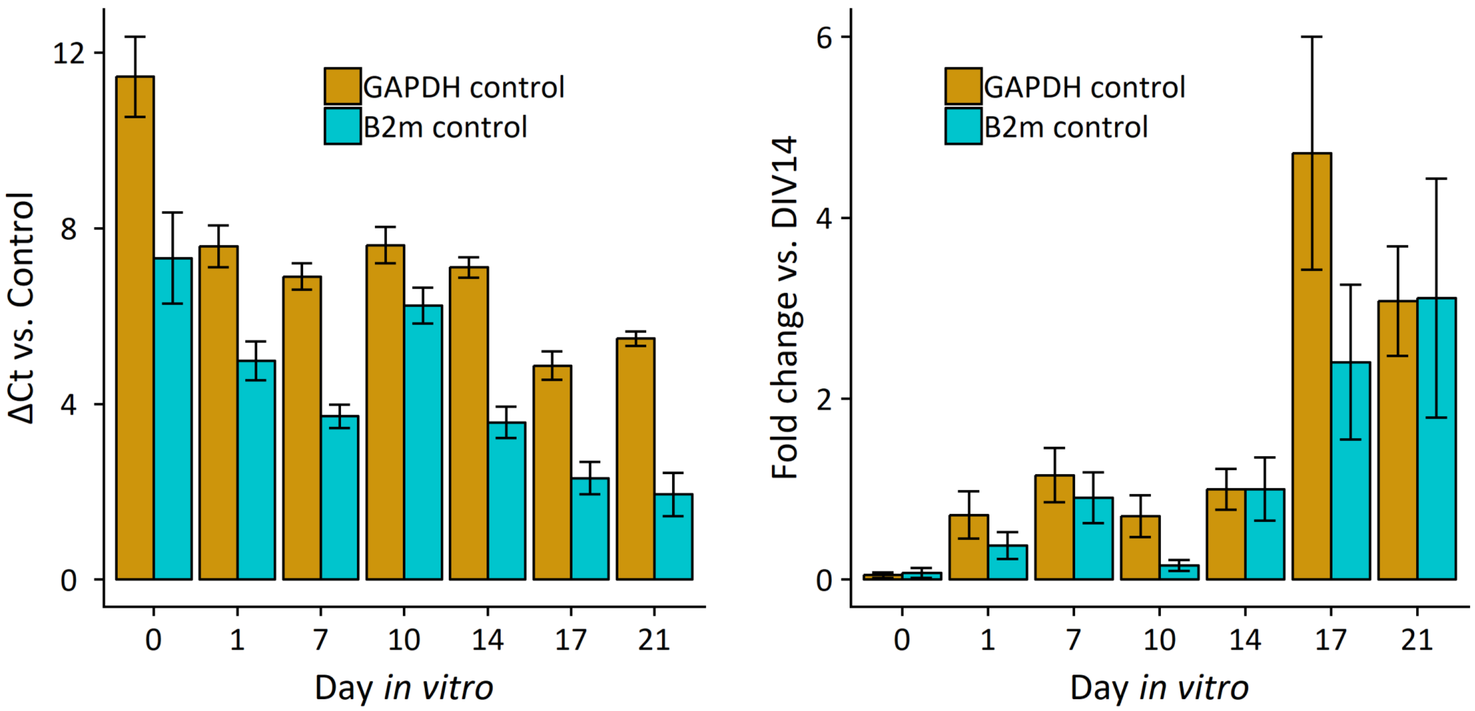

Supplemental Figure S3: RNA expression of Zn^{2+} transporter ZnT3 in cultured neurons. RT-qPCR for the ZnT3 gene *Slc30a3* in neuron cultures at day *in vitro* (DIV) 0, 1, 7, 10, 14, 17, and 21. ΔCt values were generated by normalizing threshold cycle values (Ct) to either GAPDH or beta-2-microglobulin (B2m) as internal controls. Fold changes were calculated with respect to the DIV14 timepoint. 3-4 technical replicates were run on 1 biological replicate for DIV 0, 1, 17, and 21, and 3-4 technical replicates were run on each of 2 biological replicates from 2 separate neuron preparations for DIV 7, 10, and 14. Error bars represent standard deviations, propagated from technical replicate standard deviations. Data reveal that ZnT3 mRNA is not expressed at DIV 0 but is expressed at all other timepoints tested.

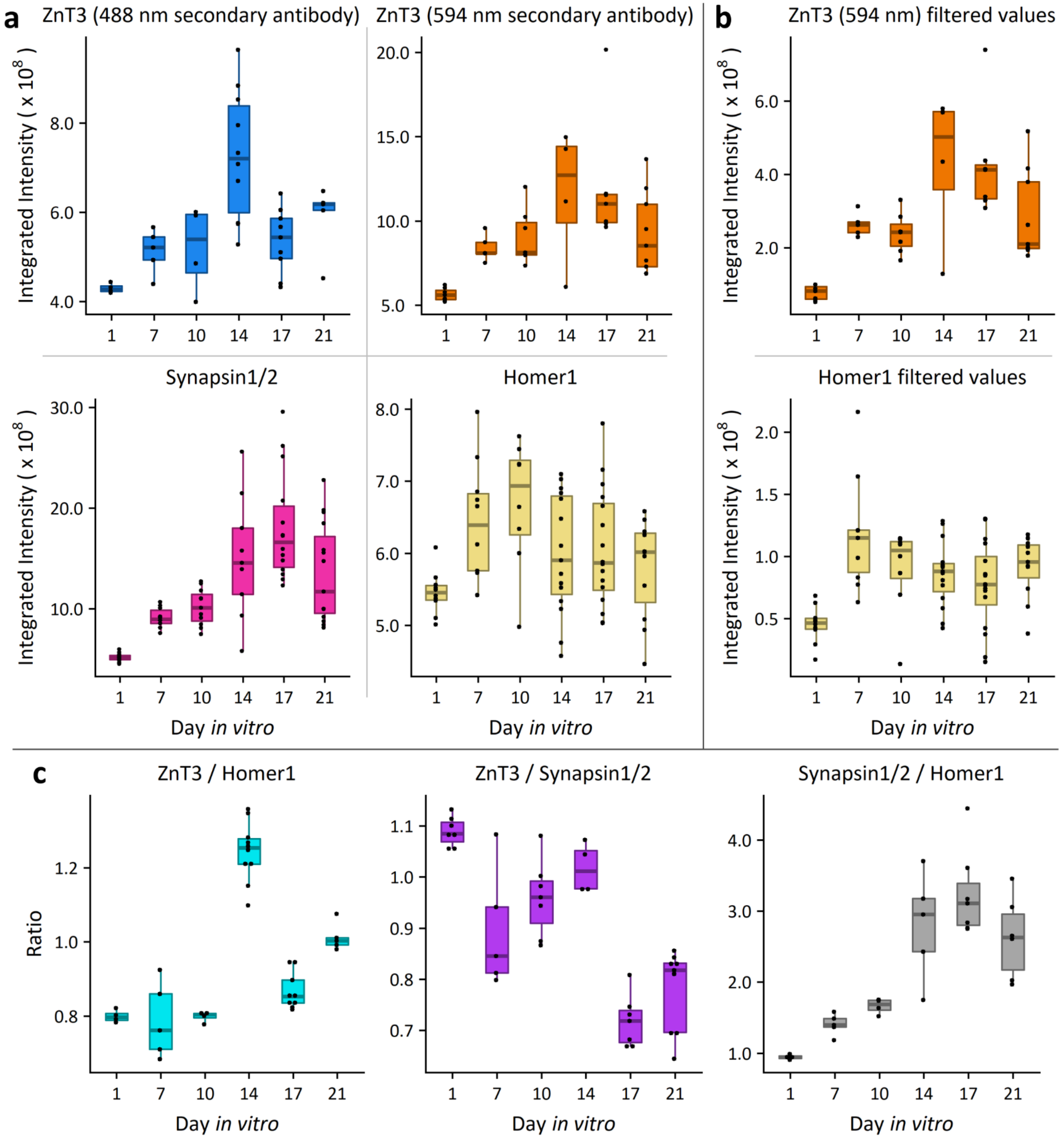

Supplemental Figure S4

Supplemental Figure S4: Quantification of immunofluorescence signal. a) Boxplots of integrated intensity measurements of full images for each separate primary/secondary antibody combination. Synapsin1/2 and Homer1 graphs include data from two biological replicates at each timepoint, whereas each ZnT3 graph includes data from one biological replicate at each timepoint, measured using the same primary antibody and different secondary antibodies. Each dot represents the integrated intensity value of one full field of view within a sample. ZnT3 and Synapsin1/2 show increased signal over time, peaking at day *in vitro* (DIV) 14. Homer1, in contrast, shows somewhat level/decreasing signal after DIV1. b) Intensity measurement trends do not change if filtering is applied to data. Adaptive thresholding was applied to images in MATLAB to filter background areas before integrated intensity calculation. ZnT3 (visualized with a 594 nm secondary antibody) and Homer1 are shown as representative examples of filtered data. Compared to the corresponding graphs in a), the spread of the data decreases, but overall trends remain the same. Replicates are the same as in part a). c) Ratio measurements comparing signal within the same field of view. Ratios indicate that fluorescent signal intensities for Synapsin1/2 and ZnT3 increase over time relative to Homer1, especially after DIV10, and that Synapsin1/2 intensity increases more substantially than ZnT3.

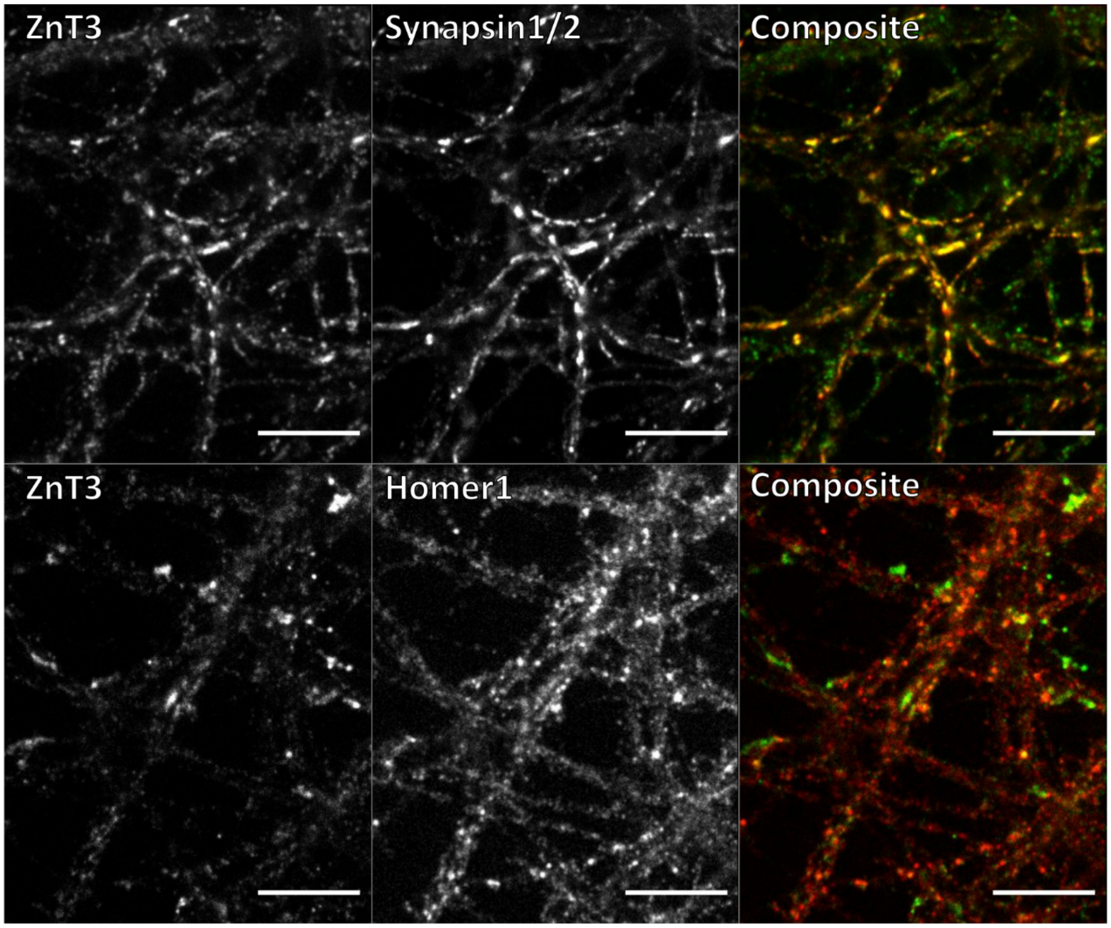

Supplemental Figure S5: Pre-synaptic localization of ZnT3. a) Immunofluorescence co-staining of day *in vitro* (DIV) 10 neuron cultures for ZnT3 (green) and pre-synaptic vesicle protein Synapsin1/2 (red) shows substantial colocalization, whereas co-staining for ZnT3 (green) and post-synaptic density protein Homer1 (red) displays little colocalization. Scale bars = 10 µm.

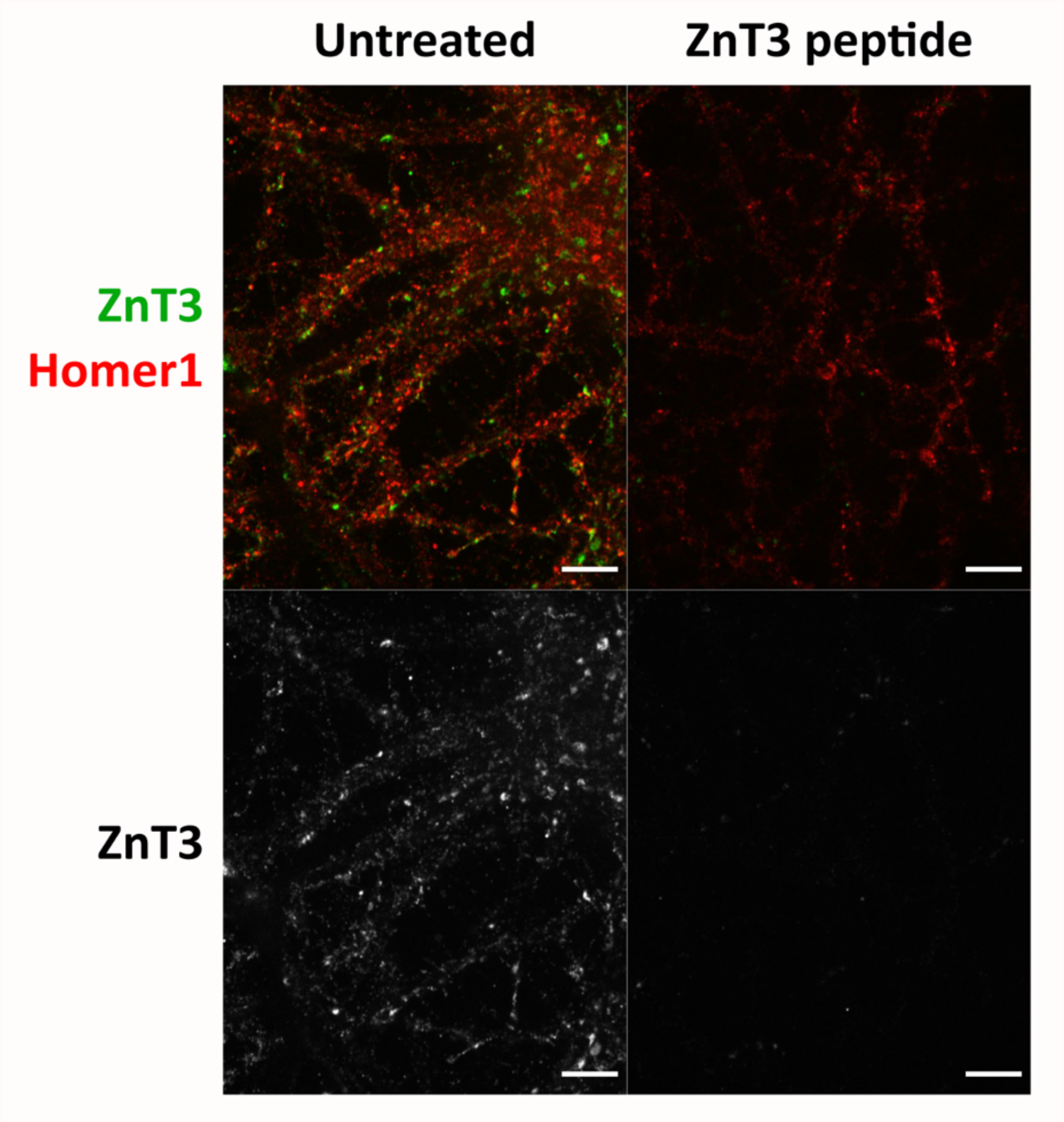

Supplemental Figure S6: Specificity of ZnT3 immunofluorescence in DIV 10 neuron cultures. For the culture displayed on the right, ZnT3 primary antibody was pre-incubated with a peptide derived from ZnT3, which abolishes most immunofluorescence signal. Individual channel intensities of both images are scaled identically. Scale bars = 10 µm.

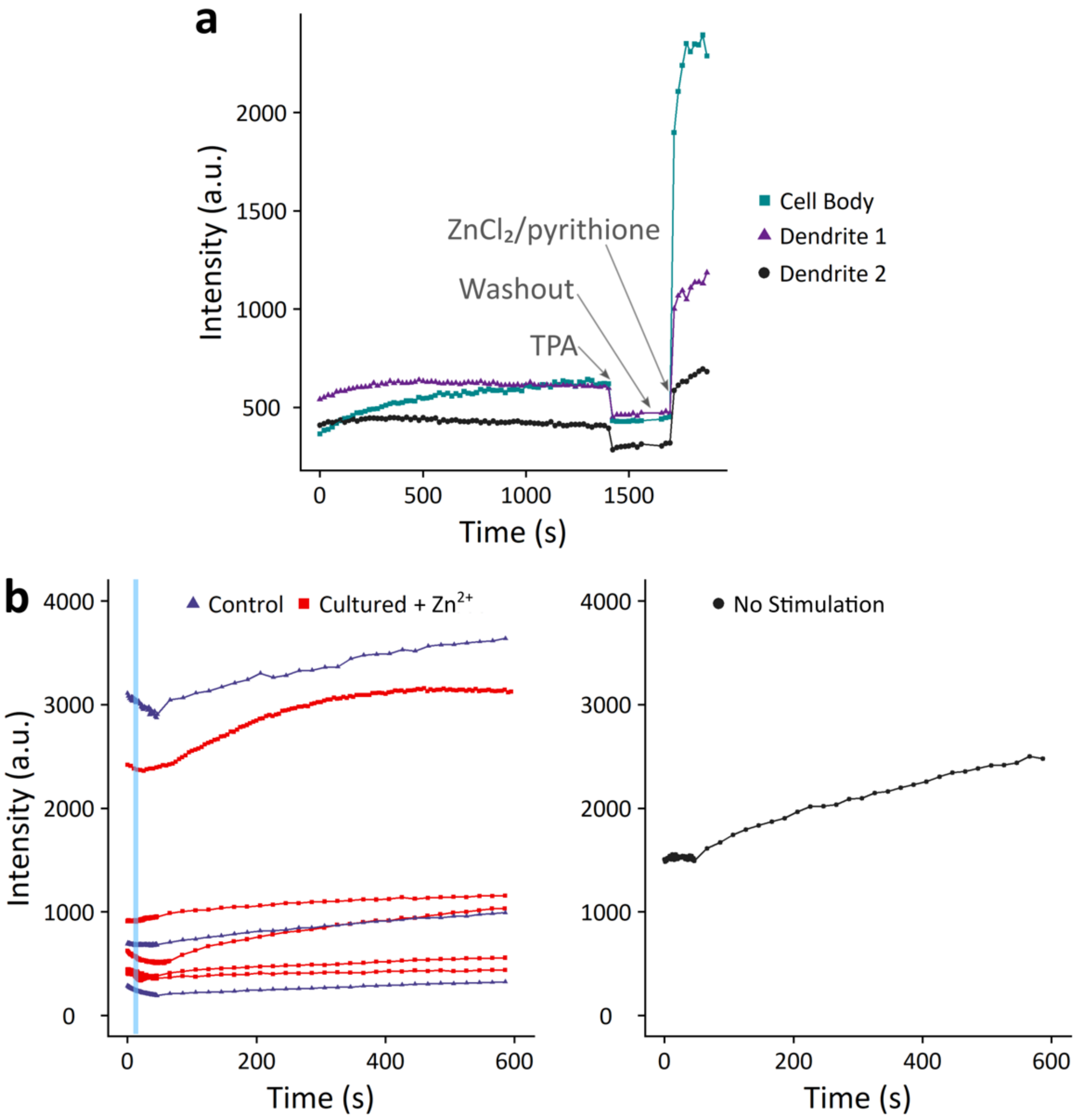

Supplemental Figure S7: Fluorescence signal of the extracellular Zn^{2+}-specific membrane dye ZIMIR. Cultures were incubated for 20 minutes with 5 µM ZIMIR prior to imaging. a) Exogenous Zn^{2+} perturbations of ZIMIR-incubated neurons. The dye is responsive to treatment with 10 µM TPA and 10 µM $ZnCl_2$, indicating that the observed fluorescence can change depending on the presence of extracellular Zn^{2+}. b) ZIMIR fluorescence upon neuronal stimulation. Cells were electrically stimulated (left panel, stimulation time indicated by blue box) or imaged without application of stimulation (right panel). Electrically stimulated cells were incubated in normal media or media containing an additional 10 µM $ZnCl_2$ for 0.5-48 hours ("Cultured + Zn^{2+}" condition). Signal is variable, but generally increases over time regardless of condition. The lack of response of ZIMIR to stimulation could be due to a lack of releasable vesicular Zn^{2+} in these cultures, or it could be due to limitations in the dye itself. For example, the K_d for ZIMIR is 450 nM[1], which would be too high to allow the dye to respond effectively to Zn^{2+} release in the low nanomolar range[2].

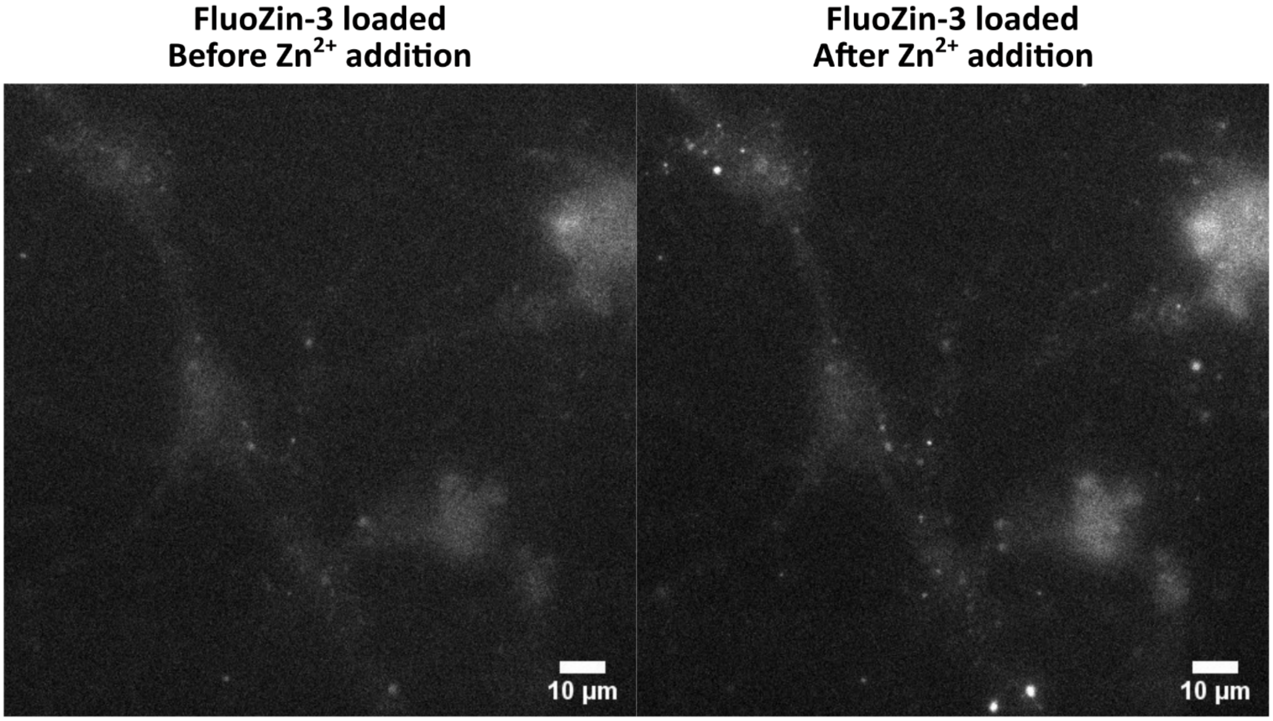

Supplemental Figure S8: Zn^{2+} treatment of FluoZin-3 loaded neurons. Neurons loaded with FluoZin-3 by electrical stimulation were imaged (left panel), then treated with 50 µM $ZnCl_2$ and imaged again (right panel). Overall signal increases somewhat, but synaptic puncta do not appear.

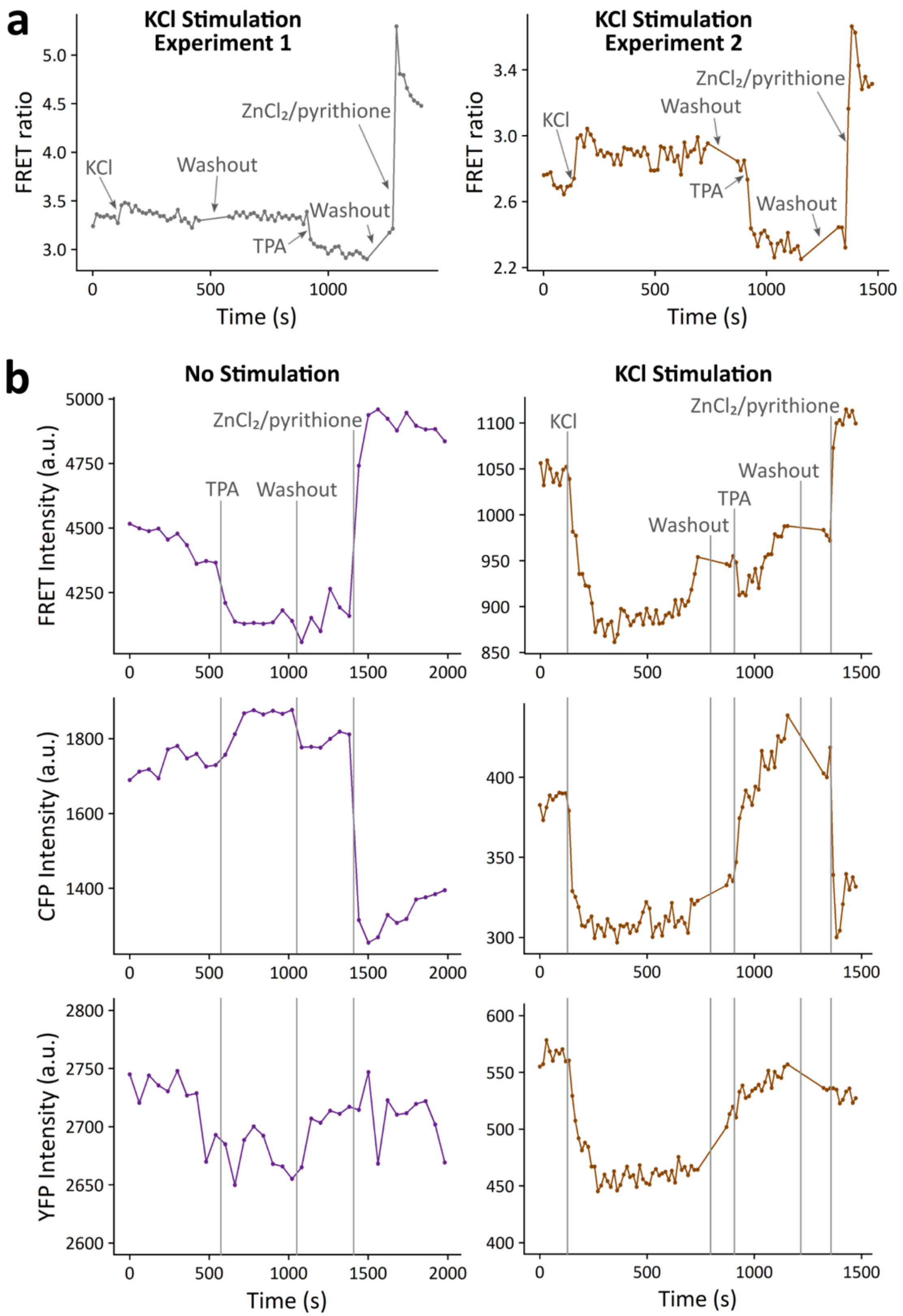

Supplemental Figure S9

Supplemental Figure S9: Zn^{2+} FRET sensor experiments in KCl-stimulated neurons. a) Two example experiments of FRET sensor recordings in neurons upon KCl treatment. Ratios correspond to the ratio of background-corrected FRET channel intensity to background-corrected CFP channel intensity in the cell body of one neuron per experiment. 50 mM KCl treatment induces a rise in FRET ratio in both experiments, and sensor in both cells subsequently responds to TPA-induced chelation of Zn^{2+} and addition of Zn^{2+}/pyrithione. b) Example intensity traces from unstimulated (resting) and KCl-stimulated neurons. With no stimulation (left), exogenous Zn^{2+} perturbations induce reciprocal responses from FRET and CFP channels and little response in YFP fluorescence excited at 514 nm, which should be irresponsive to FRET changes. Upon KCl stimulation (right), fluorescence signals from all channels drop precipitously for the duration of the stimulation, then show recovery to normal behavior. We have no data to indicate the cause of this sensor behavior, although we suspect it may be due to neuronal acidification upon KCl treatment. The FRET ratio data (a) supports FluoZin-3 AM experiments indicting an increase in cytosolic Zn^{2+} upon depolarization with KCl. However, we were concerned about the unexpected decrease in the CFP, YFP and FRET channels upon KCl treatment (b), so we chose to use FluoZin-3 AM for quantification.

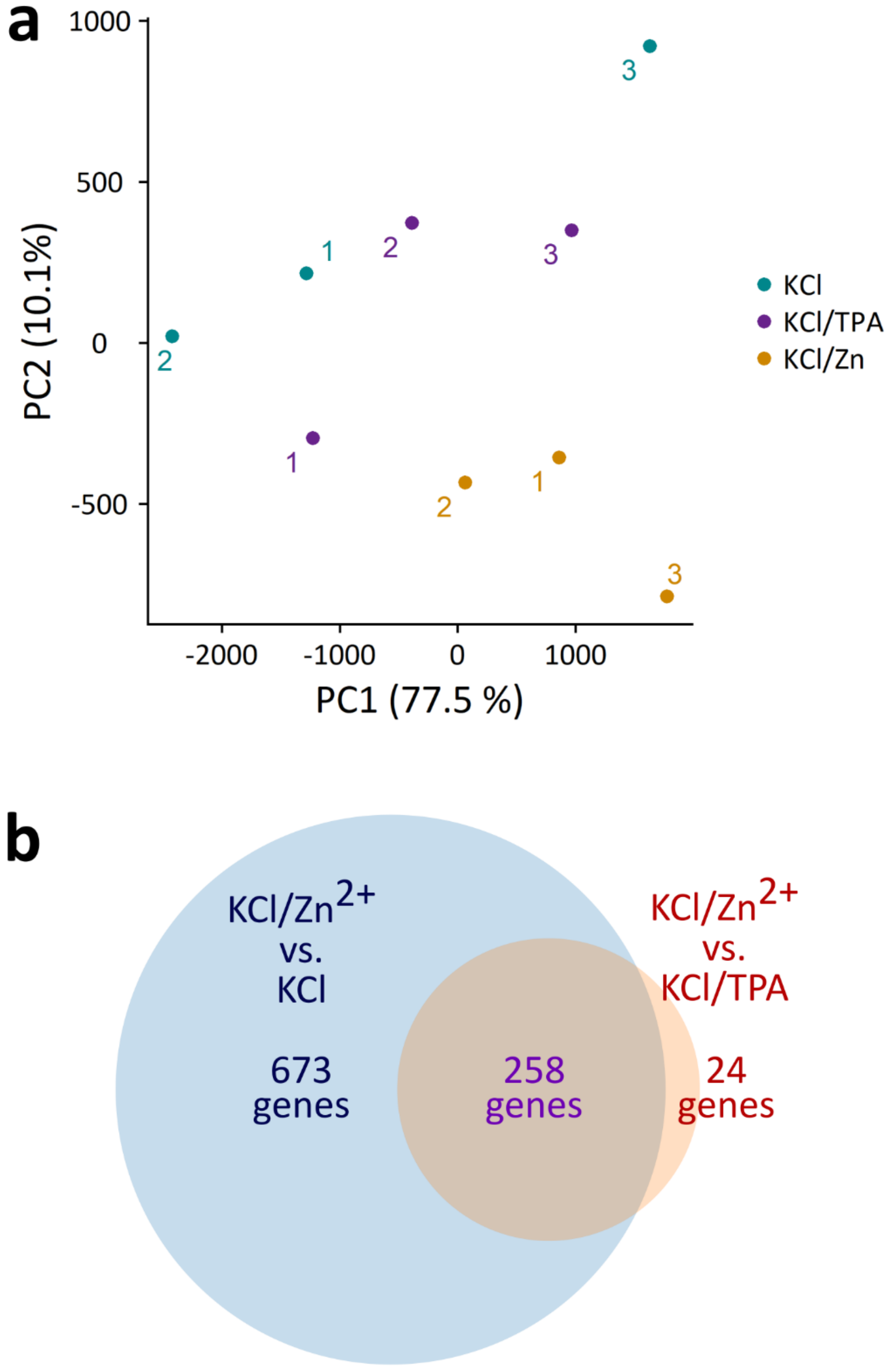

Supplemental Figure S10: Comparison of all replicates and treatment conditions in RNA-Seq data.
a) Principle component analysis of all biological replicates in each condition. PC1 and PC2 together account for 87.6% of the variability among samples. Numbers correspond to replicate designations within conditions. Samples treated with KCl or KCl/TPA generally fall on a line distinct from the clustered KCl/Zn^{2+}-treated samples. One KCl/TPA replicate shows slightly more similarity to the KCl/Zn^{2+}-treated samples, which may be a mark of heterogeneity in Zn^{2+} status. This slightly higher variability among KCl/TPA replicates also explains the fewer number of genes called significant by DESeq2 when examining the KCl/Zn^{2+} vs. KCl/TPA comparison relative to the KCl/Zn^{2+} vs. KCl comparison, even when expression trends were similar. b) Venn diagram of significantly differentially expressed genes between treatment comparisons. Differentially expressed genes were largely similar between treatment comparisons even though fewer were found to be significantly differentially expressed between KCl/Zn^{2+} and KCl/TPA treatments.

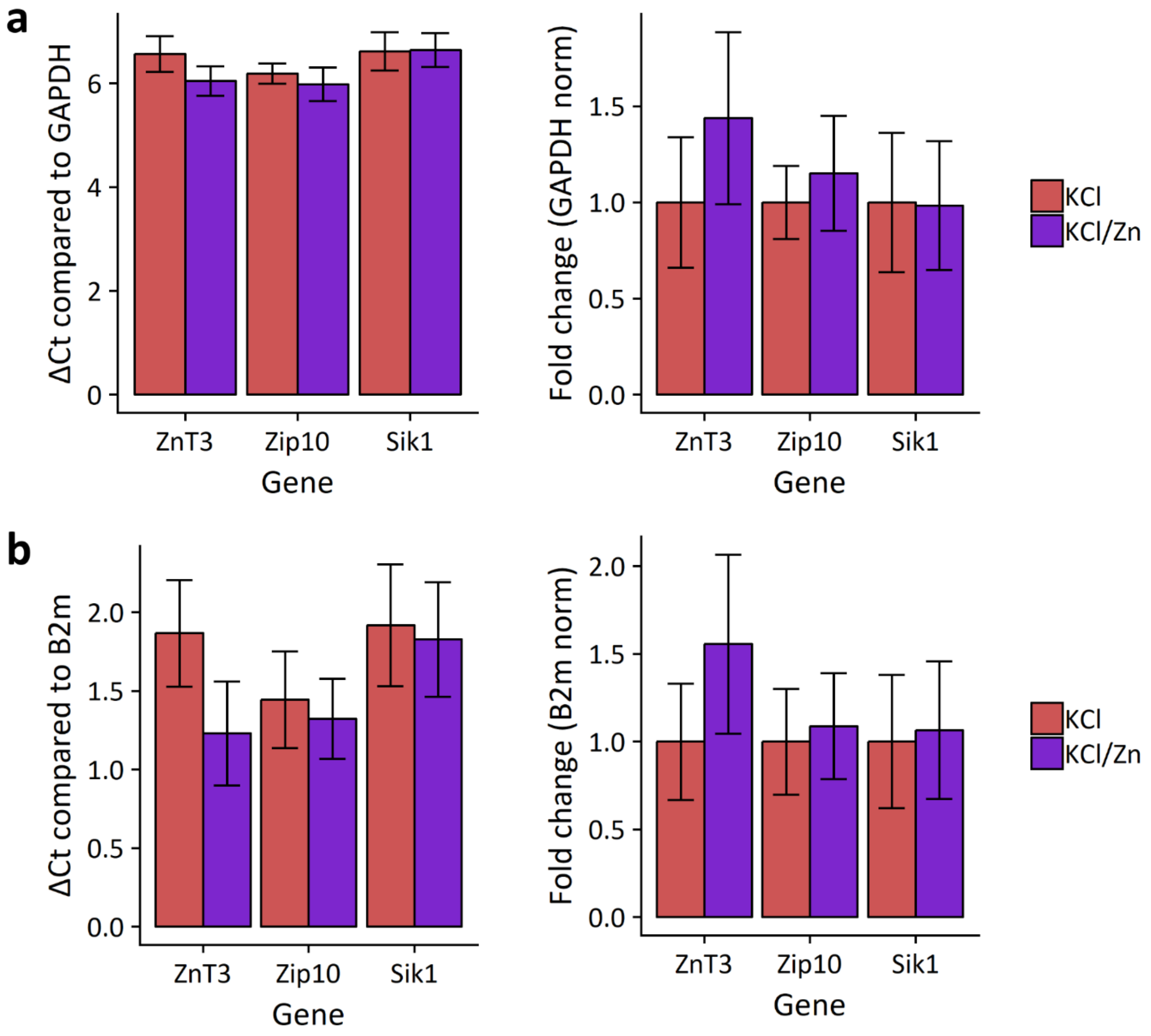

Supplemental Figure S11: RT-qPCR of select hits from RNA-Seq experiment. ZnT3 and Zip10 were observed to increase upon KCl/Zn^{2+} treatment relative to KCl treatment in RNAseq, while Sik1 was observed to decrease. ΔCt values were generated by normalizing to GAPDH (a) or B2m (b) as an internal control, then fold changes were calculated with respect to KCl treatment. Each measurement comprises 3-4 technical replicates of each of 3 biological replicates derived from one neuron preparation. Error bars represent standard deviation of the data. Although ZnT3 and Zip10 are shown to be consistent with the direction and magnitude of changes shown in the RNA-Seq data, no samples are statistically significantly different, as the magnitude of changes are small and within error. We observed no change in Sik1 by RT-qPCR. Statistical significance was assessed by unpaired two-sided t-tests for unequal variances yielding the following statistics: GAPDH normalization, ZnT3 ($t = 1.63$, $p = 0.21$), Zip10 ($t = 0.29$, $p = 0.80$), Sik1 ($t = -0.05$, $p = 0.96$); B2m normalization, ZnT3 ($t = 2.86$, $p = 0.08$), Zip10 ($t = -0.12$, $p = 0.91$), Sik1 ($t = 0.20$, $p = 0.85$).

Supplemental References

1. Li, D. *et al.* Imaging dynamic insulin release using a fluorescent zinc indicator for monitoring induced exocytotic release (ZIMIR). *Proc. Natl. Acad. Sci. U.S.A.* **108**, 21063–21068 (2011).
2. Quinta-Ferreira, M. E. *et al.* Modelling zinc changes at the hippocampal mossy fiber synaptic cleft. *J. Comput. Neurosci.* **41**, 323–337 (2016).
