# Supplementary material for: Intracellular Zn2+ transients modulate global gene expression in dissociated rat hippocampal neurons
Source: Sci Rep. 2019 Jun 28;9:9411. doi: 10.1038/s41598-019-45844-2 (PMC6598991; doi:10.1038/s41598-019-45844-2)
Supplement: Supplementary file 4 — Supplementary Dataset S1 [file 41598_2019_45844_MOESM4_ESM.pdf]

| Gene | log2(Fold change) | "False discovery rate (FDR, padj)" | Base mean expression (normalized read counts per gene) |
|---|---|---|---|
| Slc30a3 | 0.475139558 | 9.74E-09 | 1192.447638 |
| Aqp4 | 0.47067782 | 9.74E-09 | 1011.476536 |
| Tnc | 0.463139349 | 2.29E-08 | 915.7209248 |
| Fam107a | 0.441041252 | 1.71E-07 | 751.5277721 |
| Nes | 0.433657995 | 3.05E-07 | 978.6068329 |
| Tnfaip8 | 0.418916361 | 1.93E-06 | 200.1178882 |
| Il1r1 | 0.412302167 | 3.29E-06 | 376.7383423 |
| Epha7 | 0.409855283 | 3.54E-07 | 1272.096249 |
| Itpr2 | 0.403637668 | 9.67E-06 | 265.4931959 |
| Mas1 | 0.392569183 | 1.30E-06 | 1137.471759 |
| Tmem71 | 0.391684559 | 1.19E-05 | 486.829592 |
| Plcxd2 | 0.386275456 | 1.21E-08 | 2797.529581 |
| Flnc | 0.385328351 | 1.30E-05 | 575.3562198 |
| Fbln1 | 0.381451534 | 1.43E-05 | 598.5244939 |
| Rgs8 | 0.376990179 | 1.95E-05 | 794.0888349 |
| Sema5a | 0.374646028 | 8.34E-09 | 4978.082715 |
| Sema3c | 0.3741508 | 4.38E-06 | 1603.249445 |
| Ptgs2 | 0.368605083 | 2.57E-05 | 675.871117 |
| LOC100361083 | 0.367074037 | 3.57E-05 | 400.2556586 |
| Serpine2 | 0.365678915 | 1.46E-05 | 1443.13628 |
| St18 | 0.36464895 | 1.12E-06 | 3025.813261 |
| Synj2 | 0.35952824 | 2.22E-05 | 925.8229796 |
| Abca1 | 0.356191951 | 1.46E-05 | 578.7760177 |
| Rasgrp1 | 0.355479979 | 1.78E-05 | 1624.606643 |
| Vamp1 | 0.35087973 | 7.06E-07 | 2372.983515 |
| S1pr3 | 0.344827374 | 0.000221585 | 388.0047463 |
| Plec | 0.343513424 | 1.18E-05 | 2751.945593 |
| Nhlh1 | 0.343045234 | 0.000218509 | 146.5889897 |
| Oprl1 | 0.342408838 | 1.45E-06 | 1126.253072 |
| Rassf4 | 0.342104246 | 0.000160928 | 120.771047 |
| Nefh | 0.34153831 | 0.000166615 | 859.2600943 |
| Ctnna1 | 0.340062544 | 1.95E-05 | 1168.461533 |
| Penk | 0.339245293 | 0.000114294 | 783.1975357 |
| Gpr37l1 | 0.33821374 | 0.000280169 | 206.1715048 |
| Cxcl14 | 0.337584832 | 0.000310145 | 483.5943718 |
| Acsbg1 | 0.335873661 | 0.000171169 | 528.5863447 |
| Kcnj10 | 0.328783924 | 0.00044769 | 382.6172237 |
| Kcnv1 | 0.327470122 | 8.68E-05 | 827.0684487 |
| Man1a1 | 0.326450301 | 4.12E-05 | 1256.662955 |
| Igfbpl1 | 0.326019628 | 0.000484853 | 261.2644343 |
| Gja1 | 0.325421088 | 0.00033041 | 845.6271361 |
| Slc39a10 | 0.324437065 | 5.17E-06 | 3448.41086 |
| Gpr158 | 0.324143138 | 5.50E-07 | 5411.723891 |
| Tmbim1 | 0.323503474 | 0.000652218 | 236.2262795 |
| Adcy2 | 0.323352404 | 2.57E-05 | 1114.575863 |
| Abhd4 | 0.322715682 | 1.95E-05 | 1521.641582 |
| Arap2 | 0.322490239 | 0.000398728 | 372.6596849 |
| Nxph4 | 0.320682553 | 0.000741853 | 278.8816093 |

| | | | |
|---|---|---|---|
| Wipi1 | 0.319989685 | 1.95E-05 | 815.2965481 |
| Prss35 | 0.31716031 | 4.49E-05 | 89.16487042 |
| Pkp2 | 0.315264872 | 0.000959738 | 254.556821 |
| Prss23 | 0.312989885 | 0.001063078 | 223.0168885 |
| Hr | 0.310114373 | 0.000342202 | 410.6449571 |
| Pdpn | 0.310113059 | 0.001089831 | 321.6292922 |
| Csf1 | 0.309605465 | 0.001178813 | 399.0390348 |
| Chst9 | 0.308829494 | 0.000398728 | 119.6177364 |
| Nbl1 | 0.308727066 | 0.001254166 | 401.116623 |
| Tagln2 | 0.307926499 | 0.001196175 | 298.6785756 |
| Atp1a2 | 0.304037775 | 3.48E-05 | 2125.040451 |
| Tacr1 | 0.302227984 | 0.001587078 | 138.5361369 |
| Fmod | 0.301839426 | 0.001239883 | 163.0057101 |
| Synm | 0.301598198 | 9.67E-06 | 2310.023622 |
| Hhip | 0.300824879 | 0.001255129 | 250.1116072 |
| Mmd | 0.300791432 | 2.48E-06 | 14339.84114 |
| Ace | 0.300482984 | 0.001139593 | 339.8909928 |
| Itga1 | 0.299467613 | 0.000367317 | 55.50939395 |
| Fam84a | 0.299461043 | 0.000220351 | 1196.964726 |
| Lrrtm4 | 0.298741311 | 0.000746701 | 433.2799147 |
| Sstr3 | 0.298327045 | 0.000986864 | 506.5280775 |
| Ctsh | 0.297662212 | 0.001972507 | 279.2443702 |
| Gabrd | 0.297360479 | 0.002105578 | 248.3142677 |
| Mapk4 | 0.297208856 | 0.000160928 | 904.0424539 |
| Arid5b | 0.294302564 | 1.95E-05 | 917.860833 |
| Mlc1 | 0.292805664 | 0.001311582 | 800.6252248 |
| Fam129a | 0.292760548 | 0.000680557 | 87.03153846 |
| Thbs1 | 0.292305413 | 0.002494493 | 298.063122 |
| Epha4 | 0.291769557 | 6.11E-07 | 17242.0853 |
| Prkcg | 0.291744615 | 6.61E-05 | 16243.78867 |
| Aldh1a1 | 0.291106364 | 0.001587078 | 101.7289523 |
| Chn1 | 0.290155438 | 1.18E-05 | 6535.068983 |
| Sparc | 0.290112521 | 4.51E-05 | 7163.196571 |
| Anxa6 | 0.289472155 | 0.000412044 | 2739.187529 |
| Serping1 | 0.288943838 | 0.002210767 | 204.380678 |
| Alcam | 0.288272031 | 7.85E-06 | 3908.916327 |
| Hapln1 | 0.287673331 | 0.002727652 | 291.0006834 |
| Slc1a3 | 0.287448223 | 0.002686853 | 4680.084373 |
| Cav1 | 0.286641847 | 0.001119195 | 505.4249658 |
| Megf10 | 0.284737979 | 0.001787173 | 580.8062366 |
| Mt2A | 0.284724061 | 0.002864233 | 457.5409979 |
| Lrp4 | 0.284215593 | 0.003360514 | 161.3881888 |
| Pdp1 | 0.28217855 | 6.10E-05 | 1042.970185 |
| Oaf | 0.280509225 | 0.000959738 | 68.60533951 |
| Nedd9 | 0.280404397 | 0.000342202 | 1410.940282 |
| Npy1r | 0.280314952 | 0.000546485 | 994.0899232 |
| Wipf3 | 0.279558736 | 0.001101336 | 1221.760376 |
| Rnf112 | 0.27953689 | 0.000193671 | 5252.396882 |
| Thy1 | 0.279254506 | 0.003758213 | 5436.630743 |
| Ccnd1 | 0.27756801 | 0.003568054 | 736.1182005 |

| | | | |
|---|---|---|---|
| Banp | 0.27708958 | 0.002552451 | 491.8446925 |
| Ahnak | 0.276736764 | 0.001586692 | 467.0026571 |
| Rhbdf1 | 0.276658153 | 0.004712092 | 306.6719933 |
| Shisa6 | 0.276044972 | 0.000949199 | 1345.646408 |
| Sox2 | 0.27476881 | 0.004240407 | 326.4247447 |
| Fndc3c1 | 0.274766569 | 0.003842614 | 244.3328367 |
| Ros1 | 0.273056303 | 0.003581565 | 115.6389593 |
| Fgfr3 | 0.272999362 | 0.005340807 | 359.0028766 |
| Kcnab2 | 0.272723266 | 0.000636223 | 2029.431637 |
| Sorcs3 | 0.272304699 | 1.56E-05 | 3086.992175 |
| Spred1 | 0.271647536 | 0.000171169 | 1393.08714 |
| Slc15a2 | 0.271197859 | 0.003004961 | 78.21773958 |
| Gria3 | 0.271049691 | 0.002039889 | 1218.575925 |
| Chrm3 | 0.270938614 | 0.000310145 | 1370.801041 |
| Cap2 | 0.270400918 | 3.63E-05 | 3164.577969 |
| Pls3 | 0.270004217 | 0.001139593 | 933.788154 |
| Gad1 | 0.267213834 | 1.46E-05 | 12878.17868 |
| Gli3 | 0.267138901 | 0.005951774 | 131.5727379 |
| Plekhg1 | 0.266447335 | 4.42E-05 | 2153.126472 |
| Mxra8 | 0.266283818 | 0.006316149 | 119.8021389 |
| Vim | 0.265594072 | 0.006316149 | 7083.400956 |
| Nrxn3 | 0.264223553 | 0.000652218 | 4079.167762 |
| Me1 | 0.263872125 | 0.000188817 | 3122.641373 |
| Calb1 | 0.262452252 | 0.000131868 | 3309.344208 |
| Rhoc | 0.262156561 | 0.008473959 | 222.1771489 |
| Gfap | 0.260546965 | 0.007872351 | 4659.976231 |
| Hivep2 | 0.259417635 | 3.73E-05 | 20349.47801 |
| Slc6a1 | 0.259409027 | 0.00013833 | 10645.50582 |
| Plce1 | 0.259277173 | 0.00247166 | 555.4815475 |
| Kctd4 | 0.258602201 | 0.007561159 | 377.2942184 |
| Ptprg | 0.258530697 | 8.29E-06 | 5166.02607 |
| Kif1c | 0.258138012 | 0.002114059 | 1040.326935 |
| Htra1 | 0.25753098 | 0.003973489 | 77.81738929 |
| Ucp2 | 0.257185614 | 0.007872351 | 100.6774754 |
| Nefm | 0.256024679 | 0.000546315 | 16630.66103 |
| Hspa12a | 0.2556549 | 1.00E-07 | 12181.38333 |
| Gstt3 | 0.255265515 | 0.00273804 | 58.05723695 |
| Fabp7 | 0.255094082 | 0.01118857 | 2785.445833 |
| Srebf1 | 0.254923027 | 0.003983878 | 1253.273696 |
| Crhbp | 0.254876337 | 0.001586692 | 886.7681836 |
| Ppap2b | 0.25463837 | 0.003360514 | 1066.196082 |
| C1ql3 | 0.254248384 | 0.002371219 | 562.0540277 |
| Igsf11 | 0.253787796 | 0.011465658 | 295.0259606 |
| Notch2 | 0.252728949 | 0.000365851 | 1328.8438 |
| Fgfrl1 | 0.252306545 | 0.009367764 | 119.8785782 |
| Vcan | 0.251776756 | 0.001416265 | 1999.923064 |
| Nacc2 | 0.251433219 | 0.009123924 | 452.4004317 |
| Gad2 | 0.251117461 | 0.00022644 | 5247.912619 |
| Slc2a13 | 0.250530921 | 4.29E-07 | 4945.111252 |
| Hist1h2bh | 0.250273534 | 0.012629597 | 1432.957597 |

| | | | |
|---|---|---|---|
| Tnr | 0.250199641 | 0.003624003 | 1675.352834 |
| Grb14 | 0.249812635 | 0.005859516 | 451.9045659 |
| Cdkn1a | 0.249576849 | 0.005782698 | 511.7029457 |
| Grid2 | 0.24874764 | 0.01392468 | 160.1117324 |
| Htr3a | 0.248643024 | 0.013626478 | 185.8415738 |
| Hivep3 | 0.248242718 | 0.000304403 | 2117.103825 |
| Htr1a | 0.247494442 | 0.014383873 | 221.2803062 |
| Prkd2 | 0.246233147 | 0.010325575 | 484.677285 |
| Lrrc4c | 0.245678238 | 0.000232013 | 2533.997771 |
| Nab2 | 0.2453203 | 0.013309622 | 663.3031072 |
| Sspn | 0.245231995 | 0.012092199 | 96.25429945 |
| Serinc3 | 0.244393388 | 7.28E-05 | 8532.499224 |
| Gabra3 | 0.242853904 | 0.010503182 | 451.8821364 |
| Kpna2 | 0.242531338 | 0.002548495 | 1352.286925 |
| Abcb1b | 0.241922867 | 0.012602245 | 428.3994885 |
| S100b | 0.241912477 | 0.003888324 | 1195.197082 |
| Stat1 | 0.241654632 | 0.008602892 | 399.781619 |
| Lgi1 | 0.241559929 | 0.000156144 | 5248.988042 |
| Lrrn1 | 0.241419382 | 2.87E-05 | 2533.716474 |
| Ryr2 | 0.241039641 | 0.000652218 | 13374.75238 |
| Akap5 | 0.240891086 | 0.006975891 | 650.2251446 |
| Gldc | 0.240012938 | 0.018485231 | 244.7550032 |
| Lamb2 | 0.23912081 | 0.014819317 | 362.6372848 |
| Zdhhc23 | 0.238738451 | 0.017435184 | 233.2870033 |
| Ltbp1 | 0.23827589 | 0.019754558 | 197.4569728 |
| Ptk2b | 0.238272545 | 0.016040789 | 7679.919524 |
| Prickle1 | 0.237574669 | 0.000815939 | 2447.633272 |
| Enc1 | 0.234523175 | 1.96E-05 | 40377.27111 |
| Plekhg5 | 0.233559662 | 0.000941844 | 4066.87784 |
| Syt17 | 0.233491498 | 0.019669638 | 1685.221763 |
| Plekhd1 | 0.233279378 | 0.018133943 | 93.68890963 |
| Pde5a | 0.233178921 | 0.01214277 | 437.4275036 |
| Alk | 0.232941424 | 0.023458612 | 184.7767723 |
| Pragmin | 0.232486619 | 0.006898375 | 775.6385589 |
| Luzp1 | 0.232453027 | 0.001592339 | 1184.709587 |
| Reep3 | 0.232450728 | 0.017435184 | 305.7544711 |
| Mertk | 0.231877614 | 0.024512513 | 152.7888418 |
| Trak2 | 0.23151936 | 7.20E-05 | 10907.9105 |
| Slc25a18 | 0.23101858 | 0.007510619 | 68.00657054 |
| Sez6 | 0.230915796 | 0.000484853 | 24793.47722 |
| Ptprv | 0.230694771 | 0.022250269 | 153.5480913 |
| Ndrg2 | 0.230396922 | 0.025363413 | 2253.938382 |
| Tuba4a | 0.229829277 | 0.02091666 | 6990.166704 |
| Lgals3 | 0.229336957 | 0.022015994 | 136.9123645 |
| Slc1a1 | 0.229270811 | 7.37E-05 | 3144.533311 |
| Smpdl3b | 0.22835934 | 0.020433217 | 525.1962206 |
| Prrt2 | 0.227902097 | 0.000243136 | 3415.14277 |
| Chgb | 0.227542508 | 0.019921102 | 16217.12875 |
| Myo1e | 0.226968556 | 0.013165287 | 520.7121493 |
| RGD1309079 | 0.226871915 | 0.018107757 | 446.48984 |

| | | | |
|---|---|---|---|
| Slc12a4 | 0.226504257 | 0.021808237 | 305.949839 |
| Cpne6 | 0.226384084 | 0.002284859 | 3224.094925 |
| Snap25 | 0.226226859 | 1.12E-05 | 25934.17542 |
| Slitrk4 | 0.226154377 | 0.00039058 | 2087.203595 |
| Ptn | 0.226078287 | 0.008489965 | 1920.477828 |
| Gpm6b | 0.225814596 | 0.000242399 | 7734.253798 |
| Map3k5 | 0.225751984 | 0.011910377 | 524.0930797 |
| Sort1 | 0.225539666 | 1.18E-05 | 14073.42438 |
| Trim9 | 0.22465384 | 2.87E-05 | 8669.343059 |
| Gabre | 0.224370271 | 0.028218263 | 162.4247261 |
| Bmper | 0.224218125 | 0.014103773 | 63.19624302 |
| Lynx1 | 0.223840671 | 0.024912427 | 2072.964082 |
| Pon2 | 0.223401138 | 0.031846159 | 180.1238344 |
| Clstn2 | 0.223158579 | 0.02663492 | 4287.567519 |
| Spon1 | 0.22273839 | 0.023453498 | 572.9670753 |
| Dgka | 0.222709717 | 0.000654731 | 1906.583698 |
| Smc6 | 0.222565848 | 0.000416341 | 3306.883732 |
| Itgb5 | 0.222519592 | 0.032738786 | 228.5819104 |
| Vcam1 | 0.222329242 | 0.028288746 | 130.2168126 |
| Rfwd2 | 0.222265661 | 0.000527227 | 1968.547118 |
| Kcnq3 | 0.221997753 | 0.032738786 | 5104.151967 |
| Fam212b | 0.221547944 | 0.033115021 | 1027.398097 |
| Gsta1 | 0.221538162 | 0.011227678 | 938.8913309 |
| Camk4 | 0.221160331 | 0.02038614 | 932.593913 |
| Apc | 0.221129633 | 0.000652218 | 34051.19008 |
| Plp1 | 0.220959707 | 0.01964324 | 617.3248773 |
| Carhsp1 | 0.220953206 | 0.029741264 | 327.3682218 |
| Krcc1 | 0.220724349 | 0.033869211 | 199.1804368 |
| Pld2 | 0.220097052 | 0.032769233 | 146.7994864 |
| Ss18 | 0.219853725 | 0.004011882 | 982.572538 |
| Cp | 0.219813166 | 0.024452038 | 584.5745873 |
| Ephx1 | 0.218687887 | 0.015016907 | 692.4097333 |
| Fam131b | 0.218584704 | 0.000389519 | 4669.752464 |
| Slc8a2 | 0.218490922 | 0.003599623 | 8604.993812 |
| Sntg1 | 0.218369024 | 0.037234085 | 171.4679819 |
| Myadm | 0.217976592 | 0.006001738 | 9742.927649 |
| Slc16a11 | 0.217867791 | 0.037862123 | 238.7063843 |
| Caskin2 | 0.217680961 | 0.018711374 | 69.466285 |
| Cav2 | 0.217676712 | 0.027022205 | 83.65523214 |
| Gbp2 | 0.21727239 | 0.032738786 | 112.912631 |
| Il1rap | 0.217254658 | 0.018651673 | 543.4526461 |
| Lin7b | 0.216863432 | 0.026393677 | 513.9270974 |
| Arhgap25 | 0.216771628 | 0.019585331 | 73.16496519 |
| Dok5 | 0.216581567 | 0.01356512 | 1064.458017 |
| LOC100910973 | 0.216273848 | 0.03877987 | 140.0867029 |
| Slc32a1 | 0.215483799 | 0.004011882 | 3343.545779 |
| Dpy19l1 | 0.215293575 | 0.007024346 | 1050.952136 |
| Tanc1 | 0.215020523 | 0.011465658 | 1404.126743 |
| Arhgef3 | 0.214770145 | 0.008847967 | 1121.024644 |
| Stxbp6 | 0.214480153 | 0.031846159 | 131.9315772 |

| | | | |
|---|---|---|---|
| Cxxc5 | 0.213827568 | 0.003944305 | 2642.581139 |
| Adra1d | 0.2135342 | 0.021602653 | 87.90366997 |
| Sez6l | 0.213467393 | 0.000596252 | 12473.66088 |
| Crat | 0.213279584 | 0.006176282 | 3553.189728 |
| Nfe2l2 | 0.213234523 | 0.043086965 | 192.2146062 |
| Pxdc1 | 0.212858268 | 0.034290667 | 447.5408571 |
| Pde7b | 0.21249153 | 0.029741264 | 107.9833726 |
| Rhbdl3 | 0.212184967 | 0.027624449 | 751.6674476 |
| Col5a3 | 0.211472601 | 0.042688368 | 232.5818732 |
| Bend7 | 0.210992178 | 0.034313227 | 87.618403 |
| Htra4 | 0.209463065 | 0.049086524 | 1838.439788 |
| Pipox | 0.209402906 | 0.032540305 | 76.11885023 |
| Arhgap8 | 0.209398973 | 0.047556215 | 146.8580494 |
| Klhl4 | 0.209035265 | 0.029523543 | 80.04315458 |
| Cdc42ep1 | 0.208912636 | 0.025949954 | 82.29960964 |
| Kcnh7 | 0.208419832 | 0.018133943 | 882.9529901 |
| Glul | 0.208023413 | 0.00247166 | 5110.018987 |
| Jun | 0.207965776 | 0.012033282 | 3836.273524 |
| Fkbp5 | 0.207618779 | 0.011308024 | 906.747481 |
| Hspb1 | 0.207575365 | 0.007649084 | 34.70605541 |
| Zfp703 | 0.207400861 | 0.037187209 | 458.8784057 |
| Flt1 | 0.207367617 | 0.044913203 | 134.6412778 |
| Camkk1 | 0.207361122 | 0.010379596 | 2329.529214 |
| Ecel1 | 0.20734445 | 0.037862123 | 449.6650931 |
| Ankrd40 | 0.207207422 | 0.002298274 | 1403.651616 |
| Brinp1 | 0.207032382 | 0.001123988 | 5898.66025 |
| Arhgef25 | 0.206972899 | 0.040348269 | 547.4271999 |
| Tgfb1i1 | 0.206115851 | 0.029742254 | 552.4967991 |
| Plxna2 | 0.205551871 | 0.00399563 | 9342.09101 |
| Scn7a | 0.205384843 | 0.045606395 | 100.4428877 |
| Ppp3ca | 0.204566429 | 0.000484853 | 14630.37558 |
| Syt15 | 0.204399091 | 0.014383873 | 54.28131317 |
| Lrrk2 | 0.204087188 | 0.018889739 | 767.1535982 |
| Cck | 0.203909502 | 0.033033559 | 923.0091283 |
| Dgkg | 0.203888524 | 0.001081459 | 5135.94966 |
| Ephb1 | 0.203805614 | 0.023241644 | 1049.808428 |
| Prex2 | 0.203641265 | 0.039409348 | 417.9008177 |
| Fam13b | 0.203439673 | 2.04E-05 | 5470.801453 |
| Ppapdc1a | 0.202962145 | 0.035763367 | 461.8167143 |
| Fat1 | 0.202942504 | 0.005340807 | 4938.108463 |
| Cxcl12 | 0.202923231 | 0.039633774 | 497.5967031 |
| Tril | 0.202551623 | 0.037954059 | 562.6418109 |
| Grik4 | 0.202135834 | 0.021893336 | 2091.400813 |
| Pcdh20 | 0.202073197 | 0.002727652 | 4539.619311 |
| Cdk17 | 0.201996675 | 0.001254166 | 3294.195548 |
| Maob | 0.201825919 | 0.040726767 | 78.41049199 |
| Ppp1r10 | 0.201634232 | 0.002654227 | 3149.503683 |
| Myt1l | 0.20126449 | 0.000597172 | 4991.368202 |
| Itga7 | 0.201071705 | 0.048104366 | 110.8404278 |
| Ptch1 | 0.201048199 | 0.018803352 | 720.6119865 |

| | | | |
|---|---|---|---|
| Kcnip3 | 0.200809134 | 0.040208581 | 485.7154441 |
| Plcb4 | 0.200403071 | 0.016502699 | 774.4810149 |
| Klhl29 | 0.200373872 | 0.025363413 | 1293.385705 |
| Large | 0.200151576 | 0.000304426 | 6999.237826 |
| Dlgap1 | 0.19997687 | 0.001848085 | 8318.474181 |
| Ubash3b | 0.199705129 | 0.01214277 | 990.3592214 |
| Plk2 | 0.199699925 | 0.000638165 | 6018.946788 |
| Pcdh19 | 0.199309384 | 0.049877345 | 646.2985998 |
| Btbd7 | 0.199089159 | 0.029088756 | 522.8906694 |
| Fzd1 | 0.199023236 | 0.026162555 | 1729.354059 |
| Ccng2 | 0.198827908 | 0.017047352 | 1091.997632 |
| Plxdc2 | 0.198406848 | 0.035287015 | 466.3184546 |
| Syndig1 | 0.198104003 | 0.034290667 | 647.0199192 |
| Tmem63c | 0.197427787 | 0.002606092 | 2445.86478 |
| Wbscr17 | 0.197071515 | 0.003308239 | 2560.734795 |
| Arhgef2 | 0.196838334 | 0.003452381 | 2686.761875 |
| Mast3 | 0.196791141 | 0.002205977 | 12264.41489 |
| Flrt3 | 0.196681876 | 0.009873299 | 1368.731648 |
| Trib2 | 0.196664322 | 0.026306541 | 1133.02348 |
| Scn5a | 0.196566406 | 0.003624003 | 25.42992404 |
| LOC100912071 | 0.195738092 | 0.034935003 | 537.8083588 |
| Grm7 | 0.195111396 | 0.006014242 | 1360.311327 |
| Rgs16 | 0.195010374 | 0.041145662 | 93.10085579 |
| Unc5d | 0.19490417 | 0.009898909 | 1892.694417 |
| Cnr1 | 0.194786025 | 0.000446575 | 23651.22976 |
| Slc46a3 | 0.194276981 | 0.023073403 | 44.47278917 |
| Itgb4 | 0.194070037 | 0.003111068 | 26.33432449 |
| Gda | 0.193891251 | 0.00319098 | 3191.766536 |
| Zfp219 | 0.193814967 | 0.030248836 | 879.0720074 |
| Sall2 | 0.19296917 | 0.025023323 | 1115.211028 |
| Tgfb2 | 0.192460729 | 0.033705599 | 559.4923279 |
| Egr3 | 0.1923482 | 0.025391233 | 1598.653972 |
| Opcml | 0.19161648 | 0.00176041 | 7855.876607 |
| B3galt1 | 0.191018514 | 0.040329245 | 714.3058629 |
| Prom1 | 0.190283887 | 0.042270305 | 72.11308813 |
| Tp53i11 | 0.189991305 | 0.025363413 | 1838.39379 |
| Oprd1 | 0.189588392 | 0.024452038 | 47.71860274 |
| Kcnn1 | 0.189570972 | 0.037862123 | 1212.557476 |
| Nkiras1 | 0.188594549 | 0.011271677 | 1729.585672 |
| Cadm3 | 0.188495923 | 0.002114059 | 10339.2264 |
| Dmd | 0.187742474 | 0.023241644 | 867.0233897 |
| Smpdl3a | 0.187326732 | 0.042270305 | 69.28263478 |
| Nxph1 | 0.186533818 | 0.022250269 | 1335.841422 |
| Gpnmb | 0.184867545 | 0.044856602 | 1003.202244 |
| Nfat5 | 0.184532181 | 0.005885685 | 6609.085182 |
| Pde2a | 0.184473535 | 0.01246483 | 5735.744123 |
| Tspan17 | 0.184325986 | 0.010840334 | 2417.007351 |
| Mkks | 0.183938263 | 0.042270305 | 930.3443069 |
| Phyhipl | 0.183834038 | 0.000636223 | 4129.996643 |
| Ric8b | 0.183820708 | 0.044858093 | 698.6871146 |

| | | | |
|---|---|---|---|
| Ppp1r13b | 0.183669579 | 0.006049475 | 4317.196449 |
| Ppp1r12a | 0.18230688 | 0.000949199 | 4148.417977 |
| Lppr4 | 0.182034752 | 0.000649641 | 37619.57184 |
| Serpini1 | 0.181752924 | 0.000906089 | 8383.834765 |
| Arpp21 | 0.181743987 | 0.005324123 | 5447.771261 |
| Nefl | 0.18138381 | 0.006624069 | 24634.07334 |
| Phf3 | 0.180991712 | 0.002039889 | 3257.094394 |
| Grin2a | 0.180770054 | 0.007896892 | 3196.581332 |
| Limd2 | 0.180304958 | 0.031072611 | 1570.216421 |
| Glrx | 0.179736315 | 0.002250206 | 3082.338307 |
| Rasal2 | 0.179225988 | 0.027842791 | 1489.137692 |
| Zfp180 | 0.179174155 | 0.025946402 | 2126.342625 |
| Ifngr2 | 0.178972164 | 0.019669638 | 1859.214783 |
| Atp1a1 | 0.17830602 | 0.000412044 | 20202.24984 |
| Frmpd4 | 0.177258203 | 0.004262633 | 2671.200901 |
| Lrp12 | 0.17691164 | 0.006316149 | 2918.30998 |
| Mmp17 | 0.176667454 | 0.047865018 | 3093.809854 |
| Arhgap21 | 0.176636239 | 0.001101077 | 21259.41148 |
| Ppp2r2c | 0.17634854 | 0.002164604 | 6641.049318 |
| Sin3a | 0.175414124 | 0.029890229 | 1073.79101 |
| Arhgef9 | 0.175316879 | 0.002210767 | 9567.006654 |
| Zfp609 | 0.17459988 | 0.007333994 | 3687.131223 |
| Vsnl1 | 0.174403223 | 0.002727652 | 11509.29057 |
| Hace1 | 0.174385052 | 0.008526364 | 1603.661823 |
| Palmd | 0.174069359 | 0.016290986 | 2724.462011 |
| Fmnl1 | 0.173918219 | 0.011493885 | 2598.720338 |
| Tubgcp5 | 0.173412202 | 0.022010927 | 1022.680087 |
| Stxbp5l | 0.172995192 | 0.013625319 | 2183.81319 |
| Gja5 | 0.17255119 | 0.041327295 | 55.51947877 |
| Spns2 | 0.172314832 | 0.021602653 | 3829.862635 |
| Pde1a | 0.172075021 | 0.019234254 | 1567.482431 |
| Ncan | 0.171749609 | 0.006508515 | 17894.41825 |
| Slitrk3 | 0.171263933 | 0.015996949 | 1823.260568 |
| Cyld | 0.17109528 | 0.021365515 | 1570.852026 |
| Prkce | 0.171017029 | 0.015361117 | 7093.218448 |
| Bcar1 | 0.170731966 | 0.020709675 | 3723.600383 |
| Xpr1 | 0.170401198 | 0.005859516 | 3825.164762 |
| Mei1 | 0.170378424 | 0.040726767 | 60.97917292 |
| Sparcl1 | 0.170177236 | 0.003022522 | 8722.212446 |
| Nr3c2 | 0.170052081 | 0.007394237 | 5589.865788 |
| Tmem132b | 0.16991025 | 0.02772625 | 1607.84761 |
| Elmod1 | 0.169310807 | 0.004044301 | 6986.140857 |
| Igfbp5 | 0.169103797 | 0.035114799 | 1248.819131 |
| Kalrn | 0.168531586 | 0.017435184 | 9101.032242 |
| Rps6ka3 | 0.168160995 | 0.014522829 | 1766.657259 |
| Adamts4 | 0.168135179 | 0.037862123 | 43.4374774 |
| Fam20b | 0.167999324 | 0.008132983 | 4538.108243 |
| Snx5 | 0.167652272 | 0.022250269 | 1257.391199 |
| Sec14l1 | 0.167493062 | 0.010369734 | 3699.105839 |
| Camk1d | 0.166112618 | 0.01214277 | 3719.981465 |

| | | | |
|---|---|---|---|
| Dpp6 | 0.166016226 | 0.003599623 | 5826.479756 |
| Scn3b | 0.165828128 | 0.012420923 | 8290.38096 |
| Wac | 0.165322676 | 0.009837586 | 5529.195612 |
| Unc5c | 0.165316329 | 0.014046322 | 3779.822915 |
| Zdhhc17 | 0.165236523 | 0.015209229 | 2370.449228 |
| Lrrn3 | 0.164697312 | 0.005154588 | 6209.681417 |
| LOC100125362 | 0.164511065 | 0.001228498 | 7276.520231 |
| Npepps | 0.164410268 | 0.001397111 | 11164.46263 |
| Ncor2 | 0.164310825 | 0.013508163 | 10866.93435 |
| Reep5 | 0.164051092 | 0.000986864 | 11289.93669 |
| Pvrl3 | 0.16385566 | 0.018195184 | 1828.345409 |
| Itch | 0.16371116 | 0.035911336 | 1219.847184 |
| Ndrg4 | 0.162375981 | 0.002739279 | 52054.53812 |
| Kcnn2 | 0.16183055 | 0.029849073 | 1691.070618 |
| Chst1 | 0.16178798 | 0.031095491 | 6254.664317 |
| Scn1a | 0.161606253 | 0.023919479 | 2675.449346 |
| Slc23a2 | 0.161357345 | 0.001438793 | 7591.62065 |
| St3gal5 | 0.161234761 | 0.015185122 | 5928.666231 |
| Cdh10 | 0.161052907 | 0.022671852 | 1482.756267 |
| Gria1 | 0.160646021 | 0.036713912 | 9898.444758 |
| Sirpa | 0.160582598 | 0.002845863 | 6173.009236 |
| Ywhah | 0.159471924 | 0.006316149 | 45207.8422 |
| St6galnac5 | 0.158970531 | 0.033099004 | 3296.094792 |
| Susd2 | 0.158189664 | 0.014514328 | 20.5196807 |
| Zhx3 | 0.158187329 | 0.012092199 | 2683.277855 |
| Lmbrd1 | 0.157492319 | 0.015842551 | 3596.219013 |
| Mphosph8 | 0.157150686 | 0.01214277 | 2673.186887 |
| Neto1 | 0.157100881 | 0.042428917 | 3509.725129 |
| Smurf2 | 0.156814481 | 0.041241662 | 1257.590067 |
| Nedd4l | 0.156494833 | 0.00383645 | 5791.774553 |
| Pcp4 | 0.156178849 | 0.018601032 | 3896.517119 |
| Cdh9 | 0.155809969 | 0.028218263 | 2011.074371 |
| Cand1 | 0.155543214 | 0.002284859 | 8290.675361 |
| Sestd1 | 0.155102279 | 0.030623371 | 1586.639432 |
| Zfp46 | 0.15493814 | 0.027035048 | 3067.01401 |
| Tmem2 | 0.154548047 | 0.029088756 | 1765.143576 |
| Ncoa1 | 0.154532885 | 0.005023732 | 7402.14819 |
| Nap1l5 | 0.154232108 | 0.041491969 | 3095.050321 |
| Syn2 | 0.152555334 | 0.017566277 | 11095.54189 |
| Nptx1 | 0.152517754 | 0.02772625 | 18762.03088 |
| Got1 | 0.152247236 | 0.040578837 | 6117.092564 |
| Clcf1 | 0.152209681 | 0.033869211 | 32.03921787 |
| Slc35b4 | 0.152116971 | 0.02112202 | 1869.1015 |
| Kcnj6 | 0.152039444 | 0.031992794 | 6108.624105 |
| Atp2b3 | 0.151373754 | 0.024912427 | 5142.248565 |
| Klhl23 | 0.150696278 | 0.046105027 | 2838.89446 |
| Mpdz | 0.149897445 | 0.011675501 | 3191.042165 |
| Ccny | 0.149744151 | 0.035114799 | 1726.19131 |
| Sv2b | 0.149653855 | 0.012332451 | 10276.83789 |
| Ccl7 | 0.149634616 | 0.037605674 | 30.88633027 |

| | | | |
|---|---|---|---|
| Slitrk5 | 0.149192202 | 0.02137586 | 4801.014012 |
| Abi2 | 0.14903303 | 0.021698976 | 10337.63913 |
| Cyth3 | 0.148427169 | 0.018133943 | 2623.793473 |
| Tubb4a | 0.147869646 | 0.013644093 | 16180.3912 |
| Zfp148 | 0.147533233 | 0.010793125 | 3616.431404 |
| Sept5 | 0.147200444 | 0.016950655 | 17982.00783 |
| Ptprj | 0.147191177 | 0.028288746 | 4939.29719 |
| LOC690806 | 0.145316836 | 0.00758592 | 5814.366869 |
| Cadps | 0.145038997 | 0.001773769 | 8081.043592 |
| Scd2 | 0.144823697 | 0.005168369 | 72157.63094 |
| Phf20l1 | 0.144756611 | 0.048147333 | 3049.802719 |
| Map7d2 | 0.144738859 | 0.018133943 | 3545.458918 |
| Mapk1 | 0.144273172 | 0.006203705 | 24908.91226 |
| Fbxo11 | 0.144231544 | 0.01392468 | 5728.606518 |
| Pak7 | 0.144141955 | 0.021235449 | 2411.510431 |
| Atp1a3 | 0.143830701 | 0.012468676 | 75670.52699 |
| Dnajb5 | 0.143755809 | 0.038167239 | 4905.052275 |
| Tubb4b | 0.142843119 | 0.038096971 | 13515.49443 |
| Aftph | 0.14281666 | 0.013162296 | 3333.101897 |
| Ash1l | 0.142567045 | 0.04288404 | 8109.452734 |
| Kif2a | 0.142244471 | 0.014478844 | 6991.154904 |
| Tuba1b | 0.141671943 | 0.019242479 | 13689.01971 |
| Bsn | 0.141397359 | 0.043654663 | 37634.2831 |
| Ndufs1 | 0.141361782 | 0.02019948 | 4948.072436 |
| Atrn | 0.14133514 | 0.00383645 | 15772.10852 |
| Dgkb | 0.141065741 | 0.022209948 | 6308.846237 |
| Kcnq2 | 0.141001041 | 0.02137586 | 7110.704774 |
| Atp2c1 | 0.140563912 | 0.003280014 | 10098.04636 |
| Dhx9 | 0.139221885 | 0.018031318 | 7126.566752 |
| Elavl2 | 0.138915129 | 0.031324911 | 3595.840567 |
| Map1a | 0.136236133 | 0.022019662 | 39011.81027 |
| Camsap2 | 0.136197095 | 0.026658114 | 13106.17711 |
| Herc3 | 0.135814344 | 0.021887017 | 4903.782068 |
| Pea15 | 0.135596955 | 0.019234254 | 14072.13545 |
| Gabbr2 | 0.135460128 | 0.032402234 | 14292.98736 |
| Dennd5a | 0.135141637 | 0.014816594 | 8520.993386 |
| Lrfn5 | 0.134498081 | 0.04700916 | 3741.997066 |
| Jak1 | 0.134014128 | 0.003916355 | 8196.64872 |
| Dkk3 | 0.133869498 | 0.04455868 | 4735.708471 |
| Rev1 | 0.133794599 | 0.037234085 | 2556.847918 |
| Rgs7 | 0.133502817 | 0.035555212 | 3728.871427 |
| Smc5 | 0.133422446 | 0.047572876 | 2450.758329 |
| Thrsp | 0.133414894 | 0.036445519 | 25.67353828 |
| Hectd2 | 0.133134742 | 0.035911336 | 5397.555528 |
| Socs5 | 0.132760663 | 0.045209413 | 3700.131968 |
| Slmap | 0.132545755 | 0.027603195 | 3301.327518 |
| Pak1 | 0.132539731 | 0.03948995 | 6099.531246 |
| Prnp | 0.132408181 | 0.045575544 | 17020.45414 |
| Gabrb3 | 0.131744427 | 0.019521902 | 8594.68514 |
| Casc4 | 0.13149453 | 0.020647018 | 7896.475483 |

| | | | |
|---|---|---|---|
| Stx1b | 0.131166274 | 0.023831067 | 7580.138954 |
| Syn1 | 0.131119961 | 0.005782698 | 31031.66261 |
| Setd5 | 0.131022281 | 0.017092281 | 9729.200046 |
| Idh3a | 0.130537278 | 0.02994488 | 9650.632023 |
| Syt11 | 0.130411679 | 0.009946094 | 19037.80031 |
| Ei24 | 0.130041769 | 0.036156324 | 3943.478981 |
| Matr3 | 0.129956455 | 0.01957617 | 21801.02353 |
| Cnksr2 | 0.129093677 | 0.019314735 | 6369.906766 |
| Frs2 | 0.128845277 | 0.03806875 | 3821.230751 |
| Mgat3 | 0.128670755 | 0.043383214 | 5755.085099 |
| Ctnnb1 | 0.128392582 | 0.01797505 | 13404.37922 |
| Itm2b | 0.128145055 | 0.01356512 | 7888.291429 |
| Cul4a | 0.127992232 | 0.039409348 | 2785.769099 |
| Pds5b | 0.127903237 | 0.031269117 | 5383.544185 |
| Hspa4l | 0.12742128 | 0.039633774 | 4551.109153 |
| Disp2 | 0.126961916 | 0.015701317 | 18229.73253 |
| Nptn | 0.126668573 | 0.008704452 | 17024.39607 |
| Fgf13 | 0.12649576 | 0.032300228 | 14202.0198 |
| Slc35f1 | 0.12605776 | 0.021602653 | 6092.887361 |
| Csnk1g3 | 0.12347723 | 0.041772069 | 3827.511176 |
| Calm2 | 0.123081514 | 0.016084735 | 19751.91629 |
| Megf9 | 0.122873112 | 0.033674164 | 5625.783733 |
| Napb | 0.122792613 | 0.01964324 | 6095.30211 |
| Lpin2 | 0.122097639 | 0.040329245 | 4181.694533 |
| Rab6b | 0.122050331 | 0.006755664 | 54015.50188 |
| Sptan1 | 0.121681262 | 0.038167239 | 80438.5209 |
| Serinc1 | 0.121030438 | 0.032725243 | 36135.57062 |
| Fam8a1 | 0.12066188 | 0.022250269 | 4526.018802 |
| Slc24a3 | 0.120213009 | 0.038774728 | 6462.457596 |
| Olfm1 | 0.11943659 | 0.019649185 | 26959.08304 |
| Capza2 | 0.118974852 | 0.04675949 | 7332.697098 |
| Sdcbp | 0.118858139 | 0.04525858 | 12678.77421 |
| Ddx5 | 0.115864135 | 0.031773849 | 22328.56497 |
| Dnaja1 | 0.115783935 | 0.036140518 | 12200.94681 |
| Arhgap35 | 0.115225719 | 0.007896892 | 20978.23073 |
| Pja2 | 0.114919164 | 0.01280438 | 28686.84619 |
| Gtf2i | 0.113888802 | 0.031451136 | 8194.665861 |
| Nell2 | 0.112864452 | 0.024867804 | 39312.96066 |
| Cyfip2 | 0.111822693 | 0.021705245 | 24395.62578 |
| Rapgef2 | 0.111517165 | 0.042690403 | 8285.58566 |
| Synj1 | 0.110547753 | 0.034407116 | 16551.97847 |
| Nsf | 0.107411682 | 0.03960839 | 23511.06626 |
| Btbd1 | 0.106303222 | 0.049666127 | 6162.626874 |
| Epb41l1 | 0.105470418 | 0.037593247 | 20662.86823 |
| Rtn4 | 0.096876025 | 0.037187209 | 31115.08662 |
| Nfix | -0.110563022 | 0.049667608 | 12417.95849 |
| Acat1 | -0.116744033 | 0.027711072 | 6576.780296 |
| Insig1 | -0.117682311 | 0.018107757 | 15899.26348 |
| Srsf2 | -0.118181324 | 0.046487251 | 5383.602643 |
| Wdr6 | -0.118737067 | 0.006509217 | 19708.29178 |

| | | | |
|---|---|---|---|
| Iqsec3 | -0.119565899 | 0.006743086 | 19135.97203 |
| MGC109340 | -0.119807305 | 0.039696721 | 9315.239623 |
| Mllt1 | -0.120726559 | 0.029394332 | 5573.720608 |
| Dync1i1 | -0.122315747 | 0.031961947 | 8981.384964 |
| Dcaf11 | -0.123742004 | 0.026306541 | 3948.176401 |
| Ldha | -0.128147186 | 0.006392933 | 24191.84997 |
| Ftl1 | -0.129193544 | 0.020433217 | 16191.05288 |
| Dgkq | -0.129452787 | 0.037862123 | 2770.198157 |
| Mtss1l | -0.129614967 | 0.008941635 | 11049.97032 |
| Cyp51 | -0.129855477 | 0.019100745 | 21070.54808 |
| Rps14 | -0.129980798 | 0.036265139 | 2893.116292 |
| Cyb5r3 | -0.131390394 | 0.019899477 | 5739.67355 |
| Ppp1r11 | -0.131545442 | 0.044481836 | 3230.280045 |
| Narf | -0.132044219 | 0.047604542 | 4632.204724 |
| Caly | -0.132317573 | 0.022173727 | 9000.852266 |
| Tspyl2 | -0.13274872 | 0.017175598 | 4577.382181 |
| Dhcr24 | -0.133444682 | 0.020916955 | 7474.753961 |
| Aacs | -0.134128464 | 0.016386327 | 5434.349509 |
| Gpsm1 | -0.134699014 | 0.018747476 | 4438.014035 |
| Itpa | -0.134843476 | 0.029820132 | 2523.283255 |
| Psd | -0.135056387 | 0.038167239 | 12479.70467 |
| Samd4b | -0.135206931 | 0.031269117 | 5545.716765 |
| Rpl13 | -0.135499689 | 0.016741606 | 4879.646375 |
| Fcho1 | -0.135608792 | 0.024512513 | 4564.496024 |
| Sema4f | -0.136554183 | 0.038681754 | 2629.643238 |
| Fbxo33 | -0.13677771 | 0.042428917 | 2821.212711 |
| Ttl | -0.136997779 | 0.040726767 | 2543.719212 |
| Tmem35 | -0.13727865 | 0.012843999 | 19037.71954 |
| Def8 | -0.13729956 | 0.049667608 | 1675.865592 |
| Ldb1 | -0.138387866 | 0.027715469 | 2498.595066 |
| Ncln | -0.140750018 | 0.031666327 | 4251.966458 |
| Szrd1 | -0.141389878 | 0.030623371 | 1835.984844 |
| Lrrc73 | -0.141464936 | 0.042270305 | 3129.362923 |
| Fv1 | -0.141495507 | 0.014103773 | 3571.516195 |
| Flrt1 | -0.14155098 | 0.038087835 | 2167.928251 |
| Rnf40 | -0.141686328 | 0.024005584 | 2766.474983 |
| LOC689986 | -0.141814962 | 0.028223812 | 4236.781548 |
| Os9 | -0.14245177 | 0.009963459 | 4532.618141 |
| Tmem132a | -0.142788732 | 0.025863017 | 28212.87976 |
| Sipa1l2 | -0.143846684 | 0.034825167 | 3559.849951 |
| Nck2 | -0.144308673 | 0.033674164 | 3041.989438 |
| Cnrip1 | -0.145004719 | 0.006010507 | 7367.655376 |
| Zdhhc9 | -0.145348994 | 0.045606395 | 2788.149594 |
| Syt4 | -0.146149238 | 0.012092199 | 17234.58824 |
| Tra2b | -0.146436558 | 0.032738786 | 3900.987227 |
| Hmg1l1 | -0.146769006 | 0.044193656 | 1461.090655 |
| Kcnf1 | -0.146777804 | 0.040701569 | 1710.226751 |
| Rhot2 | -0.146859083 | 0.032155476 | 2841.279938 |
| Pgm2 | -0.147788699 | 0.035577319 | 2361.147748 |
| Ddx41 | -0.148014332 | 0.023453498 | 2098.00195 |

| | | | |
|---|---|---|---|
| Slc3a2 | -0.148174612 | 0.026920975 | 6530.006198 |
| Smpd3 | -0.148302547 | 0.002727652 | 8063.141022 |
| Dnajb11 | -0.149025576 | 0.013302279 | 2663.94204 |
| Lrfn4 | -0.149363197 | 0.037862123 | 4110.209538 |
| Smyd2 | -0.150283156 | 0.022780372 | 1985.916693 |
| Katnb1 | -0.150337847 | 0.02371892 | 1872.599793 |
| Dcaf15 | -0.150717524 | 0.033191583 | 1427.968526 |
| Map3k9 | -0.151786467 | 0.037674776 | 3682.188126 |
| Mrps25 | -0.15190369 | 0.045416803 | 1221.428392 |
| Gorasp1 | -0.153088008 | 0.031862741 | 1875.14548 |
| Asns | -0.153462747 | 0.047504241 | 5055.362713 |
| Lrrc16b | -0.154052609 | 0.012302923 | 7501.06349 |
| Mib2 | -0.154438709 | 0.00622509 | 5609.629034 |
| Mvk | -0.154663112 | 0.036713912 | 1203.638273 |
| Aco1 | -0.154739405 | 0.035137188 | 2667.746485 |
| RGD735029 | -0.15527484 | 0.040726767 | 2124.202208 |
| Abhd17a | -0.155286304 | 0.030623371 | 3344.219344 |
| Capn10 | -0.155602768 | 0.020711458 | 1640.832638 |
| Enthd2 | -0.156602163 | 0.017435184 | 1965.464151 |
| Slc2a8 | -0.157364769 | 0.03840161 | 1529.949015 |
| Preb | -0.157657038 | 0.014278703 | 1822.659719 |
| Hs6st3 | -0.158448318 | 0.026306541 | 1708.08383 |
| Atp13a2 | -0.158618493 | 0.003842614 | 17516.51094 |
| Xpo1 | -0.158772985 | 0.026308346 | 7434.82039 |
| Emc10 | -0.159631174 | 0.032300228 | 3502.695272 |
| Vps18 | -0.160050672 | 0.02379866 | 1451.608769 |
| Hyou1 | -0.160654579 | 0.018133943 | 10132.17874 |
| Yars | -0.161214915 | 0.005757119 | 6972.529571 |
| Rab4b | -0.161241543 | 0.024135793 | 2204.927044 |
| Marcksl1 | -0.161503485 | 0.019653023 | 3199.620277 |
| Marcks | -0.162186523 | 0.001627248 | 6429.846404 |
| Dhcr7 | -0.162351599 | 0.002606092 | 5849.399591 |
| Fam127b | -0.16246688 | 0.026529191 | 1968.310832 |
| Rdh11 | -0.163100651 | 0.029549898 | 1781.020994 |
| Sidt1 | -0.164258394 | 0.024154142 | 1585.233265 |
| Eif2ak4 | -0.164623341 | 0.044356284 | 1694.8647 |
| Sfxn3 | -0.165139586 | 0.003842614 | 6889.065994 |
| LOC100151767 | -0.166708181 | 0.029803431 | 1757.633326 |
| Fgfr1 | -0.167099153 | 0.000760744 | 18768.99173 |
| Nucb2 | -0.168332751 | 0.045017366 | 1575.931155 |
| Lss | -0.168729147 | 0.049093894 | 7529.080521 |
| Trappc9 | -0.169252006 | 0.015724316 | 1813.52055 |
| Fbxw4 | -0.170083322 | 0.026264615 | 1112.575656 |
| Manf | -0.170544042 | 0.037234085 | 1314.623272 |
| Usp2 | -0.171389674 | 0.023228425 | 1488.915097 |
| Ift20 | -0.17178581 | 0.024912427 | 1231.5298 |
| Man2c1 | -0.17241233 | 0.00562203 | 2709.178706 |
| Zfp428 | -0.172547593 | 0.039633774 | 1413.703096 |
| Efnb3 | -0.173152926 | 0.015323464 | 15159.03545 |
| Ddt | -0.173344954 | 0.022422785 | 1440.282431 |

| | | | |
|---|---|---|---|
| Prr5 | -0.173395804 | 0.026306541 | 1299.249269 |
| Sertm1 | -0.17346466 | 0.046494305 | 2196.952349 |
| Cxadr | -0.17376222 | 0.025150071 | 4772.415751 |
| Masp1 | -0.174291955 | 0.028288746 | 3178.963637 |
| Qdpr | -0.174391926 | 0.003624003 | 2216.483717 |
| Arsb | -0.174828367 | 0.032738786 | 1229.041036 |
| Adck4 | -0.174906286 | 0.03960839 | 1044.431499 |
| Lmf2 | -0.175001035 | 0.045388233 | 1047.440315 |
| Zfp414 | -0.175237127 | 0.038365243 | 761.2898953 |
| Elavl4 | -0.175415905 | 0.005951774 | 2164.659295 |
| Bbs1 | -0.175747889 | 0.00290569 | 2160.645612 |
| Rara | -0.175823164 | 0.011154981 | 1326.70723 |
| Xrcc1 | -0.175968159 | 0.017092281 | 2113.517015 |
| Sarm1 | -0.176712761 | 0.007394237 | 2657.668518 |
| Pkia | -0.17693401 | 0.003308239 | 9096.909111 |
| Vash2 | -0.177021605 | 0.035555212 | 1207.896764 |
| Tmem109 | -0.177471676 | 0.040208581 | 675.8083568 |
| RGD1306954 | -0.177497424 | 0.03207304 | 859.4668455 |
| Tbc1d8 | -0.177751547 | 0.037187209 | 982.601992 |
| Cdk5rap3 | -0.178223681 | 0.032738786 | 813.5923906 |
| Banf1 | -0.178374133 | 0.025019082 | 1367.482056 |
| Dkc1 | -0.178593481 | 0.014103773 | 1645.450232 |
| Mif | -0.178903971 | 0.003758213 | 4979.434483 |
| Rnpepl1 | -0.178992833 | 0.048767319 | 625.4348509 |
| Fdps | -0.179265535 | 0.001357429 | 13788.94263 |
| Rps6ka1 | -0.17972412 | 0.028611105 | 903.0448345 |
| Stk11ip | -0.180373513 | 0.005885685 | 1828.83039 |
| Slc2a2 | -0.180544047 | 0.034106693 | 71.81343849 |
| Dnaaf5 | -0.181551846 | 0.042431638 | 572.4544476 |
| Rnf7 | -0.181709512 | 0.037234085 | 750.0450928 |
| Oat | -0.18198256 | 0.002222624 | 3732.455834 |
| Hmga1 | -0.18241833 | 0.026264615 | 1080.050174 |
| Nup210 | -0.182519387 | 0.025950057 | 2362.293218 |
| Pxmp4 | -0.183335937 | 0.015842551 | 1125.207257 |
| Rreb1 | -0.183336497 | 0.004709091 | 3635.679917 |
| Hcn4 | -0.183611425 | 0.043159345 | 814.9062958 |
| Rps20 | -0.183685507 | 0.001848085 | 2513.509501 |
| Smyd5 | -0.183843083 | 0.037439787 | 888.1685471 |
| Cbx8 | -0.183855493 | 0.044532437 | 577.8789403 |
| Meaf6 | -0.18390788 | 0.026306541 | 1157.855806 |
| Pcsk5 | -0.184702147 | 0.011742118 | 2109.663882 |
| Nphp1 | -0.184923574 | 0.020644183 | 963.0077048 |
| Actr3b | -0.185012518 | 0.014383873 | 3114.950149 |
| Elmo1 | -0.185671243 | 0.010842481 | 2369.676789 |
| Spcs3 | -0.18604399 | 0.011200977 | 1405.410754 |
| Sec61a1 | -0.186148429 | 0.011465658 | 7083.855188 |
| Xkr6 | -0.186183515 | 0.042428917 | 608.9081495 |
| Ppdpf | -0.186205449 | 0.007818611 | 2155.412642 |
| L3mbtl2 | -0.186570357 | 0.007073671 | 1969.649582 |
| Slc7a5 | -0.186593926 | 0.025746505 | 8217.350273 |

| | | | |
|---|---|---|---|
| Chkb | -0.186971991 | 0.002727652 | 1817.474677 |
| Lmna | -0.187169179 | 0.002727652 | 3338.493224 |
| Camk1g | -0.188495271 | 0.00114407 | 3523.295723 |
| Carkd | -0.188558028 | 0.019683669 | 914.8501498 |
| Msto1 | -0.188999115 | 0.025863017 | 780.9844621 |
| Cdh13 | -0.18919545 | 0.000546315 | 10349.52773 |
| Acot3 | -0.189660228 | 0.046813713 | 482.8290788 |
| Asic2 | -0.189741601 | 0.008952814 | 3717.98889 |
| Akap17a | -0.189821973 | 0.00169723 | 1914.144057 |
| Dusp4 | -0.189923026 | 0.001311582 | 5145.952515 |
| Sdr39u1 | -0.190173808 | 0.006377434 | 1327.630156 |
| Gpr19 | -0.190505616 | 0.041441588 | 554.6865046 |
| Ntng2 | -0.190723439 | 0.001119195 | 6328.864021 |
| Usp36 | -0.190782263 | 0.018107757 | 2351.076513 |
| Ier2 | -0.190807458 | 0.020647018 | 967.5433943 |
| Sorcs2 | -0.190826722 | 0.000790766 | 14296.15996 |
| Fam214b | -0.19142512 | 0.010376237 | 1169.113428 |
| RGD1562310 | -0.191442033 | 0.046006438 | 1225.592495 |
| Ablim3 | -0.191446632 | 0.022602741 | 2357.211263 |
| Rbm3 | -0.191697897 | 0.000324575 | 6470.632477 |
| Gpd1l | -0.19171841 | 0.00758592 | 1474.085964 |
| Emx2 | -0.191864009 | 0.016898182 | 900.306781 |
| Tchp | -0.191988346 | 0.033722222 | 560.2892048 |
| Foxp4 | -0.192964407 | 0.031269117 | 604.7074637 |
| Tle2 | -0.192979711 | 0.009873299 | 1110.354615 |
| Acat2 | -0.192992587 | 0.001415587 | 5094.636155 |
| Cstf2 | -0.193014508 | 0.003973489 | 9476.421144 |
| Commd4 | -0.193063257 | 0.003888324 | 1336.433484 |
| Trim67 | -0.193805341 | 0.028765717 | 982.1116548 |
| Col7a1 | -0.194325096 | 0.038096971 | 61.33143049 |
| Rasl11b | -0.194580878 | 0.017955801 | 3246.260482 |
| Ccdc64 | -0.195362617 | 0.001416265 | 2755.826652 |
| Mob3a | -0.195741344 | 0.01214277 | 1058.782488 |
| Ubxn8 | -0.195857384 | 0.048767319 | 392.8390031 |
| Mmp24 | -0.195907551 | 0.002548495 | 7653.764753 |
| Sec24d | -0.196396511 | 0.014274816 | 1925.116426 |
| Inpp4b | -0.196581685 | 0.049890964 | 558.9146342 |
| Frmd6 | -0.196704067 | 0.044728247 | 532.6994759 |
| Stk40 | -0.197009471 | 0.001597734 | 2492.451556 |
| Pafah1b3 | -0.197226426 | 0.030427729 | 741.1107438 |
| Kiaa0895l | -0.197548281 | 0.000280169 | 4387.311518 |
| Itfg2 | -0.198281463 | 0.038313379 | 393.4827854 |
| Chst11 | -0.198589444 | 0.005306701 | 1129.208721 |
| Mrpl54 | -0.198834226 | 0.026572065 | 542.2630052 |
| Ccnd3 | -0.198872547 | 0.006001738 | 1303.342845 |
| Brd9 | -0.199106476 | 0.003984275 | 3685.02162 |
| Osbpl7 | -0.199188074 | 0.012749658 | 992.3139921 |
| Mafk | -0.199703005 | 0.018133943 | 668.9037489 |
| Tmem184b | -0.199793309 | 0.002039889 | 3333.171727 |
| Ptp4a3 | -0.200014893 | 0.006975891 | 1158.062511 |

| | | | |
|---|---|---|---|
| Adck5 | -0.200143225 | 0.017566277 | 713.7550481 |
| Sept6 | -0.200685043 | 0.001941944 | 2858.374807 |
| Ogfod2 | -0.200965285 | 0.041572563 | 406.6506856 |
| Ppp2r3b | -0.201261101 | 0.020625384 | 581.2433248 |
| Pak6 | -0.201330501 | 1.46E-05 | 10202.93101 |
| Midn | -0.201514661 | 0.002436608 | 3127.762647 |
| Lrrc20 | -0.201655255 | 0.040726767 | 351.639929 |
| Uck1 | -0.202156185 | 0.003074395 | 1431.892414 |
| Ntsr1 | -0.20241224 | 0.037187209 | 432.3545427 |
| RGD1311946 | -0.203460308 | 0.003714202 | 1601.531235 |
| Ddah2 | -0.204321278 | 0.015695664 | 865.1468937 |
| Aldh2 | -0.205016034 | 0.013719224 | 697.9749779 |
| Adamts15 | -0.205036063 | 0.02994488 | 504.6807148 |
| Slc16a13 | -0.206260763 | 0.039724849 | 312.2795162 |
| Arnt | -0.206385663 | 0.016898182 | 854.8029212 |
| Hdac5 | -0.206495942 | 7.85E-06 | 7890.490885 |
| RGD1564149 | -0.206643008 | 0.010379596 | 41.69968992 |
| Nr4a2 | -0.206652833 | 0.016502699 | 4286.891831 |
| Rell2 | -0.207804727 | 0.000204269 | 3273.094129 |
| Rgs9 | -0.208046723 | 0.043578428 | 398.5167937 |
| Alkbh7 | -0.208110218 | 0.03960042 | 377.2968187 |
| Per1 | -0.208135742 | 6.80E-05 | 5236.680247 |
| Mcm5 | -0.208157511 | 0.028969763 | 70.52304839 |
| LOC100911483 | -0.208244633 | 0.001899058 | 1454.165552 |
| Stac2 | -0.20842067 | 0.013625319 | 969.6880509 |
| Hexdc | -0.20864382 | 0.007698632 | 1289.061061 |
| Mrpl23 | -0.209867007 | 0.031181184 | 426.1640242 |
| Cirbp | -0.210122643 | 0.00273804 | 2532.117226 |
| Hmgn2 | -0.21024844 | 0.013162296 | 3643.579564 |
| Tenm3 | -0.210519343 | 0.013760079 | 4314.183822 |
| Tmem242 | -0.210847219 | 0.014383873 | 620.1027215 |
| Eln | -0.210963292 | 0.041640941 | 196.3027036 |
| Lef1 | -0.211240935 | 0.033475251 | 467.717839 |
| Dnaaf2 | -0.211464943 | 0.012843999 | 835.1668403 |
| Eml2 | -0.211670618 | 0.000264131 | 5262.66834 |
| Lime1 | -0.212437431 | 0.019242479 | 936.6177593 |
| Tfr2 | -0.212977499 | 0.043706542 | 241.0457746 |
| Npas2 | -0.213427915 | 0.002369065 | 1365.459463 |
| Scpep1 | -0.213784958 | 0.002120398 | 1300.474667 |
| Hcn3 | -0.215047918 | 0.002250206 | 2165.473934 |
| Hes6 | -0.21518026 | 0.000534567 | 2547.846058 |
| Gpr88 | -0.215358743 | 0.021594925 | 570.9437371 |
| Sema3a | -0.215479311 | 0.037234085 | 302.2500113 |
| Klhdc8b | -0.215814437 | 0.002336462 | 1796.740672 |
| Gle1 | -0.216159584 | 0.010457903 | 658.0566415 |
| Pla2g4b | -0.216630195 | 0.024452038 | 602.1985367 |
| Angel1 | -0.217001165 | 0.018133943 | 809.3068029 |
| Dusp1 | -0.217110032 | 0.038337659 | 2269.677968 |
| Slc6a11 | -0.217795648 | 0.008633047 | 2041.187543 |
| Hadh | -0.21892057 | 0.025863017 | 393.3813968 |

| | | | |
|---|---|---|---|
| Cdc14b | -0.21948727 | 0.036142433 | 183.7453137 |
| B3gnt7 | -0.219533633 | 0.029088756 | 105.4845423 |
| Ick | -0.219963578 | 0.002444179 | 1825.237897 |
| Srrd | -0.220195336 | 0.014715631 | 500.1472424 |
| Csrnp1 | -0.220349571 | 0.005808726 | 1171.458682 |
| Kif13a | -0.22050031 | 0.003360514 | 2559.332476 |
| Crh | -0.220699308 | 0.005101587 | 63.38862689 |
| Snapc1 | -0.22073983 | 0.025863017 | 349.7787989 |
| Rpp21 | -0.221530606 | 0.021602653 | 399.402146 |
| Stac3 | -0.222471074 | 0.012617795 | 91.56365627 |
| Zdhhc4 | -0.223404392 | 0.011772009 | 492.071969 |
| Psat1 | -0.224856256 | 0.006001738 | 1492.997303 |
| Rfx5 | -0.224981958 | 0.006898375 | 1475.334403 |
| Padi2 | -0.225275392 | 0.026658114 | 312.5155573 |
| Rasd2 | -0.225309139 | 0.000398728 | 4864.19585 |
| Sfxn4 | -0.225317229 | 0.024912427 | 396.4758787 |
| Trim66 | -0.22545421 | 0.012092199 | 640.9720931 |
| Il6r | -0.226822988 | 0.000790766 | 1570.922549 |
| RGD1308134 | -0.227173882 | 0.012501191 | 567.7421525 |
| Atf5 | -0.227740385 | 0.007986809 | 1757.395429 |
| Ppp2r3a | -0.22813542 | 0.010760035 | 1661.291414 |
| Acaa1a | -0.22918768 | 0.004779033 | 1079.709976 |
| Sesn2 | -0.229948086 | 0.00399563 | 4221.887056 |
| St3gal1 | -0.230232838 | 0.017435184 | 474.6394108 |
| Fhod3 | -0.230363301 | 0.007279112 | 1335.001511 |
| Pde8b | -0.230667289 | 0.011675501 | 445.1923249 |
| Vegfa | -0.230742871 | 0.001476652 | 6997.175487 |
| Qsox1 | -0.23153802 | 0.001360801 | 1293.292907 |
| Rapgef3 | -0.231666466 | 0.021172303 | 365.3229607 |
| Gys1 | -0.232488109 | 0.00273804 | 1231.376247 |
| Lgi2 | -0.232580172 | 0.020647018 | 4443.071685 |
| Pcdh8 | -0.233137962 | 1.19E-05 | 14576.58915 |
| Ece1 | -0.233172997 | 0.002438336 | 1948.004032 |
| Calb2 | -0.233665068 | 0.018485231 | 595.8373326 |
| Slc7a8 | -0.233862852 | 7.19E-05 | 4785.489966 |
| Eefsec | -0.234455947 | 0.009367764 | 441.015876 |
| Sult2b1 | -0.234504918 | 0.010672682 | 507.5982948 |
| Rtkn2 | -0.234878551 | 0.021235449 | 144.557764 |
| Fam213a | -0.235111206 | 0.000171169 | 2243.509627 |
| Cep89 | -0.235477505 | 0.00268119 | 924.7888818 |
| Slc17a6 | -0.237727125 | 0.00516921 | 1689.182537 |
| Tmem151b | -0.238730109 | 3.48E-05 | 2986.424127 |
| Hsd17b10 | -0.238738188 | 0.002816022 | 638.9931662 |
| Leng1 | -0.238964206 | 0.013625319 | 349.3941106 |
| Slc26a6 | -0.239820082 | 0.002548495 | 728.490878 |
| Arhgdib | -0.240334662 | 0.018682314 | 188.6029068 |
| Cflar | -0.240573302 | 0.004262633 | 694.5461305 |
| Cars2 | -0.240616264 | 0.008078978 | 492.6759878 |
| Acta2 | -0.242483346 | 0.014103773 | 169.7679179 |
| Chodl | -0.242647057 | 0.00987797 | 103.0262314 |

| | | | |
|---|---|---|---|
| Mylk | -0.242873597 | 0.011321492 | 551.3400964 |
| Plxnd1 | -0.244491546 | 0.000191433 | 3272.018751 |
| Gcat | -0.244701705 | 0.007377942 | 526.5657014 |
| Pycr1 | -0.247456299 | 0.011193098 | 451.5366342 |
| Wfs1 | -0.24786643 | 0.003360514 | 2152.930051 |
| Adamts18 | -0.248265035 | 0.006203705 | 971.6864208 |
| Grem2 | -0.248428885 | 0.002727652 | 688.9773725 |
| Tox | -0.248558968 | 0.01398471 | 340.6966842 |
| RGD1563348 | -0.249861369 | 0.001623728 | 954.6037603 |
| Podxl2 | -0.249898917 | 8.29E-06 | 6356.98046 |
| Grip2 | -0.249966453 | 0.006602808 | 619.3459787 |
| Pdxk | -0.250034694 | 0.000586251 | 1099.550566 |
| Fam107b | -0.250924998 | 0.00383645 | 872.8770074 |
| Nfil3 | -0.250928971 | 0.001750898 | 3625.938557 |
| Hsd17b7 | -0.251171224 | 0.002548495 | 1564.642416 |
| Kcnk3 | -0.251178643 | 0.006439344 | 355.1881694 |
| Ndrg1 | -0.252085791 | 0.004044993 | 1088.076688 |
| Arid5a | -0.25271094 | 0.002727652 | 843.6318161 |
| Sdf2l1 | -0.252784653 | 0.01214277 | 160.5646015 |
| Triobp | -0.25278735 | 0.004240407 | 731.6101023 |
| Myof | -0.253355827 | 0.003963291 | 1520.351461 |
| Gpc2 | -0.254500677 | 0.011321492 | 174.7472328 |
| Ppp1r15a | -0.254944185 | 7.45E-05 | 2374.156559 |
| Galt | -0.254962875 | 0.011313193 | 187.5153716 |
| Ankrd44 | -0.255530485 | 0.004069515 | 1057.313878 |
| Gm2a | -0.255563091 | 0.000654731 | 1174.398623 |
| Slc25a1 | -0.255730725 | 0.00010236 | 3317.484611 |
| RGD1312005 | -0.256956633 | 0.001554886 | 1238.236306 |
| Dcn | -0.25750334 | 0.005410668 | 815.234834 |
| Npr3 | -0.260402667 | 0.00758592 | 177.1883866 |
| Bace2 | -0.260741737 | 0.002598327 | 756.6836332 |
| Nkd2 | -0.26170455 | 0.006267496 | 346.3941424 |
| Gch1 | -0.262014988 | 0.005324123 | 145.4957673 |
| Sapcd2 | -0.262145182 | 0.008456706 | 179.0622427 |
| Popdc3 | -0.26311401 | 0.007024346 | 245.0036204 |
| Stat5b | -0.265871998 | 0.000939987 | 768.6051844 |
| Mvd | -0.265936188 | 1.30E-05 | 5554.860037 |
| Trib1 | -0.266438043 | 0.001239883 | 857.8521998 |
| Crem | -0.26659047 | 0.001005537 | 793.3791445 |
| Plagl1 | -0.268429399 | 0.002548495 | 1730.429979 |
| LOC691153 | -0.268739961 | 0.003848642 | 703.2379643 |
| Fam213b | -0.271959226 | 0.003624003 | 377.1517883 |
| Lpin3 | -0.273511997 | 0.005008161 | 237.4688015 |
| Wdr63 | -0.274855668 | 0.005138474 | 217.9736708 |
| Prrt4 | -0.27524002 | 0.001077181 | 651.7773875 |
| Pycr2 | -0.275588611 | 0.000786668 | 546.0111107 |
| Fam184b | -0.276435039 | 0.000243136 | 1179.414226 |
| Anks6 | -0.278063421 | 0.001139593 | 572.8278015 |
| Chst8 | -0.27868095 | 0.001140101 | 663.5881162 |
| Fxyd6 | -0.279320956 | 0.000406397 | 3037.299641 |

| | | | |
|---|---|---|---|
| Fyb | -0.279588764 | 0.002731893 | 129.9391462 |
| Xylb | -0.279917774 | 0.000966156 | 1628.708914 |
| Dusp5 | -0.28063319 | 1.86E-05 | 1565.328605 |
| Yif1a | -0.280973665 | 0.00273804 | 281.279439 |
| Kif26a | -0.281079133 | 0.00273804 | 355.8959631 |
| Fam163b | -0.28368748 | 0.000279846 | 1471.427817 |
| Rbm11 | -0.285839081 | 0.000846465 | 537.3931964 |
| Rnf152 | -0.286839922 | 0.001896337 | 721.5876847 |
| Rcan2 | -0.288943675 | 0.002787057 | 4843.507141 |
| Fchsd1 | -0.290139903 | 0.000652218 | 725.8925563 |
| Fam46a | -0.291289346 | 0.00254699 | 144.651318 |
| Tmem163 | -0.293888935 | 0.00069968 | 1452.510202 |
| Etv1 | -0.294248781 | 2.58E-06 | 3057.471707 |
| LOC499781 | -0.295551986 | 0.00222858 | 185.5680812 |
| Chn2 | -0.297751823 | 0.000275985 | 1154.360664 |
| Hdac11 | -0.298056029 | 0.00010236 | 1388.623042 |
| Il3ra | -0.29915161 | 0.000356778 | 658.3031545 |
| Cort | -0.307081912 | 0.001255129 | 199.573852 |
| Rasip1 | -0.308561603 | 0.000232158 | 758.0603055 |
| RGD1310769 | -0.309027665 | 3.21E-05 | 2235.076835 |
| Hebp1 | -0.309263423 | 0.000796572 | 327.7390582 |
| Rgs6 | -0.312079489 | 0.000941844 | 412.5388685 |
| Scarf2 | -0.315247743 | 0.000717163 | 285.2182528 |
| Snx8 | -0.318217595 | 0.000148329 | 497.7414144 |
| Adcyap1 | -0.319921738 | 0.00010236 | 817.1034269 |
| Scly | -0.324599813 | 2.57E-05 | 654.8930901 |
| Creg1 | -0.32536733 | 2.82E-05 | 1235.874134 |
| Nexn | -0.327881859 | 0.000170926 | 843.8472542 |
| Maff | -0.345781947 | 0.00022644 | 204.9295121 |
| Epha10 | -0.373745051 | 1.46E-05 | 514.4357831 |
| Lgr5 | -0.383027426 | 2.02E-05 | 171.8139932 |
| Sik1 | -0.459470757 | 8.34E-09 | 1542.23405 |
| Tpbg | -0.464807337 | 5.50E-09 | 1877.34536 |
